# Defining species-specific and conserved interactions of apical membrane protein 1 during erythrocyte invasion in malaria to inform multi-species vaccines

**DOI:** 10.1007/s00018-023-04712-z

**Published:** 2023-02-27

**Authors:** Damien R. Drew, Danny W. Wilson, Gretchen E. Weiss, Lee M. Yeoh, Isabelle G. Henshall, Brendan S. Crabb, Sheetij Dutta, Paul R. Gilson, James G. Beeson

**Affiliations:** 1 Burnet Institute, 85 Commercial Road, Melbourne, VIC 3004 Australia; 2 Research Centre for Infectious Diseases, School of Biological Sciences, The University of Adelaide, Adelaide, 5005 Australia; 3 Departments of Medicine, Microbiology and Immunology, and Infectious Diseases, The University of Melbourne, Melbourne, 3010 Australia; 4 Central Clinical School and Department of Microbiology, Monash University, Victoria, Australia; 5 Walter Reed Army Institute for Research, Silver Spring, USA

**Keywords:** *Plasmodium falciparum*, *Plasmodium vivax*, Antibodies, RON2, Peptides, Therapeutics, Inhibition

## Abstract

*Plasmodium falciparum* and *P. vivax* are the major causes of human malaria, and *P. knowlesi* is an important additional cause in SE Asia. Binding of apical membrane antigen 1 (AMA1) to rhoptry neck protein 2 (RON2) was thought to be essential for merozoite invasion of erythrocytes by *Plasmodium* spp. Our findings reveal that *P. falciparum* and *P. vivax* have diverged and show species-specific binding of AMA1 to RON2, determined by a β-hairpin loop in RON2 and specific residues in AMA1 Loop1E. In contrast, cross-species binding of AMA1 to RON2 is retained between *P. vivax* and *P. knowlesi*. Mutation of specific amino acids in AMA1 Loop1E in *P. falciparum* or *P. vivax* ablated RON2 binding without impacting erythrocyte invasion. This indicates that the AMA1–RON2-loop interaction is not essential for invasion and additional AMA1 interactions are involved. Mutations in AMA1 that disrupt RON2 binding also enable escape of invasion inhibitory antibodies. Therefore, vaccines and therapeutics will need to be broader than targeting only the AMA1–RON2 interaction. Antibodies targeting AMA1 domain 3 had greater invasion-inhibitory activity when RON2-loop binding was ablated, suggesting this domain is a promising additional target for vaccine development. Targeting multiple AMA1 interactions involved in invasion may enable vaccines that generate more potent inhibitory antibodies and address the capacity for immune evasion. Findings on specific residues for invasion function and species divergence and conservation can inform novel vaccines and therapeutics against malaria caused by three species, including the potential for cross-species vaccines.

**Supplementary Information:**

The online version contains supplementary material available at 10.1007/s00018-023-04712-z.

## Introduction

Malaria is a major global health problem with > 240 million cases and > 600,000 deaths reported annually [1]. Despite initial gains in reducing malaria in the early 2000s, progress has stalled since 2015 and the burden is now increasing. Furthermore, the impacts of climate change are expected to substantially exacerbate malaria in coming years [2]. As such, new interventions including novel vaccines and therapeutics are urgently needed. While *Plasmodium falciparum* accounts for the majority of malaria-induced morbidity and mortality worldwide [1], around 3 billion people are also at risk of *P. vivax* malaria globally and there is a growing recognition that it is also a major cause of severe malaria and chronic illness [3–6]. Even in the absence of exposure to infectious mosquitoes, dormant liver stage hypnozoites can release infectious *P. vivax* merozoites (which infect erythrocytes) into the bloodstream resulting in repeated relapses over time and chronic illness. *P. knowlesi* is closely related to *P. vivax* and a significant cause of human malaria in Southeast Asia, occurring as a zoonotic infection from macaque monkeys [7]. The leading vaccine against *P. falciparum*, RTS,S vaccine, has modest efficacy (30–50%) against *P. falciparum* only [8], and progress on *P. vivax* vaccines has been very limited, with extremely few candidates in development or clinical trials [9, 10]. The development of vaccines or novel therapeutics that protect against the blood stage of *P. falciparum* and *P. vivax* is a priority because this is the life stage during which clinical disease occurs. Targeting merozoites, which infect erythrocytes, prevents blood-stage replication and disease, and specifically targeting merozoites would prevent relapses of *P. vivax* [11].

*P. falciparum* and *P. vivax* are thought to have independently evolved from primate to human hosts [12, 13] and have adopted alternative erythrocyte invasion mechanisms, ligands and receptors [10, 11]. Because *P. knowlesi* is phylogenetically closely related to *P. vivax*, it is a valuable model of *P. vivax*, a species that cannot be maintained in long-term culture [14]. Apical membrane antigen 1 (AMA1) is one of few invasion proteins shared between *P. vivax* and *P. falciparum* and has been shown to play an essential role in infection of host erythrocytes by merozoites of *P. falciparum* [15]. AMA1 is also a prominent target of acquired immunity and has been a leading vaccine candidate [11, 16–19]. Furthermore, AMA1 is present in all mammalian and avian *Plasmodium* species, even though most other merozoite invasion ligands and surface proteins are different between species [11]. The three-domain structure of AMA1 is conserved among species, but the amino acid sequence varies [20–23]. Immediately before merozoite invasion, AMA1 binds to rhoptry neck protein 2 (RON2), an event that initiates formation of the tight junction, essential for host cell invasion [23, 24].

RON2 is a parasite-derived protein that is released by merozoites, and it translocates into the host erythrocyte membrane (forming a complex with RON4, 5 and 8) [25, 26]. A short β-hairpin loop in the C-terminal domain of RON2 is exposed on the erythrocyte surface and interacts with the hydrophobic groove of AMA1 [23, 27]. This molecular event has been primarily established for *P. falciparum*, but less clearly for *P. vivax*. Synthetic peptides corresponding to this loop of *Pf*RON2 bind to *Pf*AMA1 and inhibit *P. falciparum* invasion, leading researchers to conclude that this interaction is essential for invasion [15, 23, 26–29]. Agents that inhibit the AMA1–RON2 interaction in *P. falciparum* effectively disrupt erythrocyte invasion [30], making it an attractive target for prophylactic and therapeutic interventions.

We recently reported that *Pv*AMA1 can functionally complement *Pf*AMA1 in *P. falciparum* parasites. *P. falciparum* parasites with genetic replacement of *Pf*AMA1 with *Pv*AMA1 had no apparent changes in erythrocyte invasion dynamics or replication rate [31]. This observation indicated conservation of function between *Pf*AMA1 and *Pv*AMA1, and raised the question of whether *Pv*AMA1 might have the ability to bind *Pf*RON2 protein during erythrocyte invasion since AMA1-RON2 binding was considered essential for invasion, or that there may be additional AMA1 binding interactions during invasion. The establishment of *P. falciparum* parasites expressing *Pv*AMA1 and techniques to mutate *Pf*AMA1 and *Pv*AMA1 sequences [31, 32] provided a powerful platform to investigate the function of AMA1 and RON2 in invasion. Furthermore, this model provides a rare opportunity to use transgenic approaches to introduce targeted mutations to understand the function of an invasion ligand of *P. vivax*. Currently, knowledge of specific differences or common features in the function and interactions of *P. falciparum* and *P. vivax* invasion ligands is extremely limited, even for leading vaccine candidates where there are often major differences between these two most important malaria species.

In this study, we investigated the functional conservation and divergence of AMA1 and RON2 with *P. falciparum, P. vivax* and *P. knowlesi* to gain insights for vaccine and therapeutic development for these three major causes of human malaria. *P. vivax* cannot be readily propagated or genetically manipulated in vitro; therefore, we used a chimeric approach of expressing *Pv*AMA1 in *P. falciparum* parasites, which we have previously validated [31]. *P. knowlesi* is closely related to *P. vivax* and is also used as a model of *P. vivax* [33]. We used complementary approaches to advance our understanding of AMA1 in erythrocyte invasion by *P. falciparum, P. vivax* and *P. knowlesi* including, (i) evaluating invasion-inhibitory RON2 peptides with *P. falciparum, P. vivax* and *P. knowlesi* parasites, (ii) generating transgenic parasites with mutation of the RON2-binding regions (Loop1E) of AMA1 and evaluating their invasion function and (iii) evaluating the impact of mutations in AMA1 on susceptibility to invasion-inhibitory antibodies.

## Methods

### Ethics statement

For generating antibodies, animal ethics approval was provided by the Animal Ethics Committee of the Walter and Eliza Hall Institute (Australia), or the SVL Animal Care and Use Committee (for Quadvax, Fig. 7). No human samples or data were used.

### Role of funding sources

Funders had no role in study design; collection, analysis and interpretation of data; writing of manuscript; and in the decision to submit the paper for publication.

### Sequence analysis

Amino acid sequences were aligned in Geneious (https://www.geneious.com), and a distance matrix constructed in R using DECIPHER [34]. A neighbour-joining dendrogram was created using default settings, and a heatmap constructed with SPIDER [35]. The dendrogram was rendered using FigTree (http://tree.bio.ed.ac.uk/software/figtree), and images were further edited with Inkscape (http://www.inkscape.org).

### Parasite culture and genotyping

*P. falciparum* asexual stage parasites were maintained in culture in human erythrocytes (blood group type O +) at a haematocrit of 4% in RPMI-HEPES supplemented with 0.25% (w/v) Albumax™ (Invitrogen) and 5% (v/v) heat inactivated human serum. *P. falciparum* were synchronised using sorbitol and heparin treatments as described previously [36, 37]. *P. knowlesi* [14] was maintained in RPMI-HEPES with 0.5% v/v Albumax II (Gibco) and synchronised using Percoll. Transgenic W2mef *P. falciparum* strains expressing *Pf*AMA1 W2mef allele (W2-W2), *Pf*AMA1 3D7 allele (W2-3D7) and *Pv*AMA1 Palo Alto allele (W2-*Pv*AMA1) were generated in previous studies [31, 32, 38].

### Expression of AMA1 proteins in HEK293F cells

#### Sequence selection and modification

The protein sequences for each of the antigens were selected: *Pf*AMA1 (XP_001348015.1; 3D7 genotype), *Pv*AMA1 (ACB42433.1; Palo Alto genotype) and *Pk*AMA1 (XP_002259339.1; Strain H) (Supplementary data Table S1). At the N-terminus of the sequences, the native signal peptide was removed and replaced with a signal peptide for tissue plasminogen activator, followed by a 6-histidine tag. At the C-terminus of all sequences, the sequence was truncated to remove the transmembrane domain and cytoplasmic tail. All sequences were assessed for potential glycosylation sites (http://www.cbs.dtu.dk/services/NetNGlyc/). The AMA1 sequences for each species were then modified to prevent potential glycosylation (*Pf*AMA1, six changes; *Pv*AMA1, three changes; *Pk*AMA1, seven changes) (Supplementary data Table S2).

#### Generation of protein expression vectors

Each AMA1 protein sequence was used to generate DNA sequences that were codon-optimised for mammalian expression and then synthesised (GeneArt). The final protein sequences are provided (Figure S7). Synthetic genes were supplied in a pUC vector, and then cloned into pcDNA 3.1(+) using *Xho*1 and *Bam*H1 restriction sites. The final plasmids were quantified and used to transfect HEK293F cells.

#### HEK293F cell culture and transfection

HEK293 Freestyle™ cells (Thermo Fisher Scientific) were cultured following the manufacturer’s protocols and used for expression of recombinant proteins as described [39, 40]. In brief, cells were cultured in Erlenmeyer shaker flasks (125 mL, Corning) with FreeStyle™ 293 Expression Medium (Thermo Fisher Scientific) at 37 °C, 8% CO_2_ at 135 rpm on an orbital shaker. Cells were counted using the trypan blue (0.4%; Thermo Fisher Scientific) cell exclusion method using Countess™ Cell Counting Chamber Slides (Thermo Fisher Scientific) and the Countess™ automated cell counter (Thermo Fisher Scientific). HEK293F cells were transfected for protein expression following the manufacturer’s protocol (Thermo Fisher Scientific) with minor alterations. On the day of transfection, cells were centrifuged (700* g* at 4 °C, 10 min) and resuspended in HEK293F expression media with 1:100 antibiotic/anti-mycotic solution (Thermo Fisher Scientific) to a final density of 1 × 10^6^ cells/mL. For a 30 mL transfection, 90 µL of polyethylenimine transfection reagent (25 kDa linear; Polysciences; stock 1 mg/mL) was added to 0.6 mL of OptiPro™ Serum Free Medium (Thermo Fisher Scientific) and incubated for 5 min. This solution was then added to the DNA solution (30 µg of purified plasmids and 0.6 mL of OptiPro™ Serum Free Medium (Thermo Fisher Scientific)). After 10 min incubation at room temperature, this final solution was added to the cells, and then returned to the orbital shaker and incubator. The next day, lupin (1:40 of 20% w/v, Biotech Solabia) and pluronic acid F-68 (1:100 of 10% w/v, Thermo Fisher Scientific) were added. The expressed proteins were harvested 6 days post-transfection by centrifuging cells (700* g,* 10 min) to collect supernatant that was then filtered (0.2 µm membrane) and stored at 4 °C until purification. Protein expression was tested using SDS-PAGE gels and western blot.

#### Protein purification and dialysis

The harvested media containing the expressed proteins was passed over nickel resin columns (Life Technologies), washed with 20 mM imidazole/phosphate-buffered saline (PBS) (Sigma-Aldrich), and bound proteins eluted in several 1 mL fractions in 500 mM imidazole/PBS. Eluted fractions were tested for the presence of protein by spectroscopy and SDS-PAGE. Fractions containing protein were pooled, filter-sterilised, dialysed into sterile PBS, and adjusted to a concentration of 1 mg/mL by centrifugation using 10 000 MW cut-off filters.

#### SDS-PAGE, colloidal staining and western blot analysis of recombinant proteins

The folding and purity of expressed proteins were tested by colloidal blue staining and western blot. For colloidal blue stained gels, 1–2 µg of protein was run on an SDS-PAGE gel under either native or denaturing conditions (+ TCEP, to break conformation-specific disulfide bonds), and then stained with the colloidal Coomassie stain (GelCode Blue Stain Reagent, Thermo Fisher Scientific). For the western blot, 50 ng samples were run on an SDS-PAGE gel under either native or denaturing conditions (+ TCEP, to break conformation-specific disulfide bonds) and then transferred to nitrocellulose membrane (iBlot Transfer Stack nitrocellulose, Thermo Fisher Scientific). Membranes were then probed with antigen-specific antibodies: 4E11 mAb against *Pf*AMA1 and rabbit polyclonal antibodies against *Pv*AMA1.

### Peptides

We used two sets of RON2 peptides: (i) peptides of 39 amino acids, based on the *Pf*RON2sp1 peptide originally described by [22]: *Pf*RON2 (Asp-2021 to Ser-2059), *Pv*RON2 (Asp-2050 to Thr2088) and *Pk*RON2 (Asp-1975 to Thr-2013); (ii) peptides of 48 amino acids, based on the *Pf*RON2L peptide described by [29] (Asp-2021 to Lys-2067) but with the addition of an N-terminal Met-2020: *Pf*RON2L (Met-2020 to Lys-2067) and *Pv*RON2L (Met-2049 to Lys-2096). All RON2 peptides were synthesised with cyclised cysteine residues to create the RON2 loop. We found that the shorter peptides were water soluble and more suitable for testing in GIA. The longer peptides based on the *Pf*RON2L peptide were used to dissect the functional roles of the N- and C-terminal amino acids flanking the β-hairpin loop core. However, these peptides needed to be solubilised in DMSO, making them less suitable for use in invasion and growth inhibition assays. As a negative control we used a small extracellular sequence of *Pf*RON3 protein (Glu-574 to Lys-603) (ERYGVLKQCPLDIVKNLNQQCEYVSFEIKK), synthesised as a peptide of 30 amino acids with cyclised cysteine residues. All peptides were synthesised by LifeTien (Somerset, NJ, USA).

### Antibodies and RON2L binding assays

The generation of rabbit antibodies against recombinant ectodomain *Pf*AMA1 (3D7 allele) and *Pv*AMA1 (Palo Alto allele) proteins has been described previously [32, 41]. mAbs were isolated from mice vaccinated with *Pf*AMA1, as reported previously [41]. Standard ELISA was used to confirm antibody specificity using established methods [16, 42]. RON2L binding to recombinant AMA1 proteins was measured by a plate-based assay. Briefly, recombinant AMA1 proteins or the control Pfs230 protein were coated onto 96-well ELISA plates (Maxisorb) at a concentration of 10 μg/mL, overnight at 4ºC. Plates were washed and incubated with 10 μg/mL biotin-tagged *Pv*RON2L peptide at 37ºC for 1 h. Plates were then washed and incubated with 2 μg/mL streptavidin-conjugated HRP (Millipore) for 1 h at 37 °C. After washing, plates were developed by the addition of ABTS.

### Generation of transgenic parasite lines

Genes encoding *Pf*AMA1 (3D7 allele) and *Pv*AMA1 (Palo Alto allele) with mutated RON2-binding residues (AMA1-M) were generated by Spliced by Overlapping End (SOE) PCR, using a previously described codon-optimised *Pf*AMA1 and *Pv*AMA1 DNA as templates and synthetic oligonucleotides incorporating relevant DNA mutations (Geneworks Australia) [31, 32, 38]. All mutated *Pf*AMA1 and *Pv*AMA1 alleles were inserted into the plasmid vector pCC1AMA1TP.1 and transfected into W2mef parental *P. falciparum* as previously described [31, 32, 38]. *P. falciparum* populations confirmed to have plasmid integration into the AMA1 target after positive selection with WR99210 were cloned by limiting dilution, and positive populations were confirmed by integration-specific PCR.

### Live-cell microscopy

Highly synchronous parasite cultures at 4% haematocrit were diluted 1/25 in media and allowed to settle to produce a monolayer of cells in a 35 mm Fluorodish (World Precision Instruments). All live-cell experiments were performed at 37 °C on a Zeiss AxioObserver Z1 fluorescence microscope equipped with humidified gas chamber (94% N_2_, 1% O_2_ and 5% CO_2_) [24]. Time-lapse videos were recorded at four frames per second with a high-resolution AxioCam MRm camera. ImageJ and Prism (GraphPad) were used to perform image and statistical analyses. For data sets with normal distribution an unpaired *t* test was used. For data sets without normal distribution the Mann–Whitney *U* test was used. For comparison of deformation scores between groups a Chi-square analysis was performed. A value of *p* ≤ 0.05 was used as the determinant of statistical significance for all tests.

### Quantifying invasion inhibition

Growth-inhibition assays were performed over two cycles of replication and measured by flow cytometry as described in detail elsewhere [31, 32, 43, 44]. Synchronised early-trophozoite stage *P. falciparum* were grown with rabbit or human IgG at a concentration specified in each assay. *Pf*RON2 and *Pv*RON2 peptides were solubilised in PBS and tested against PBS controls. *Pf*RON2L, *Pv*RON2L, *PfPv*RON2L and *PvPf*RON2L were solubilised in neat DMSO, then diluted to a final concentration of 2 mg/mL peptide in 10% DMSO in PBS; in these experiments an equal volume of 10% DMSO in PBS was used as the control. After two invasion cycles, early-trophozoite stage *P. falciparum* were stained, and parasitaemia measured by flow cytometry. Each sample was run in either duplicate or triplicate in at least two separate assays for each AMA1 transfectant line. Percentage invasion inhibition was calculated as:

 = (1-(parasitaemia in test well ÷ mean parasitaemia in non-inhibitory control wells)) × 100.

Non-immunised rabbit IgG or KLH-immunised rabbit IgG were tested as controls in invasion inhibition assays when evaluating rabbit IgG. *Pf*RON3 peptides were used as controls when evaluating testing peptides. Non-immunised mouse IgG was used as controls in assays evaluating mouse monoclonal antibodies. RON2 peptides and antibodies to AMA1 have been previously shown to specifically inhibit merozoites invasion of erythrocytes [24, 37]; therefore, inhibition of growth by RON2 peptides and anti-AMA1 antibodies was interpreted as inhibition of invasion.

### Polyacrylamide gel electrophoresis (SDS-PAGE) and western blot analysis

SDS-PAGE and immunoblot analysis of parasite proteins was performed as previously described [31]. Synchronised schizont-stage parasite cultures were lysed with saponin, washed in PBS and resuspended in non-reducing SDS sample buffer (Invitrogen). Samples were sonicated and heated to 100 °C for 5 min prior to SDS-PAGE. Proteins were separated on 3–8% tris–acetate gels (Invitrogen) and transferred onto nitrocellulose using the iBlot system (Invitrogen) according to standard protocols. Blots were probed with anti-3D7, anti-*Pv*AMA1 [31] or QuadVax rabbit IgG (20 μg/mL IgG) or 2.5 μg/mL 1F9, 4E11, 4E8, 1B10, 5A6 or 1E10 monoclonal antibodies [41]. Blots were also probed with a mouse monoclonal antibody generated against the *P. falciparum* HSP-70 protein (1:2000) as a loading control. Horseradish peroxidase-coupled (HRP) goat anti-rabbit Ig (1:2000) or sheep anti-mouse Ig (1:2000; Millipore) were used as secondary antibodies.

### Parasite multiplication assays

The multiplication rate of transgenic parasites expressing either wild-type (WT) 3D7 AMA1 (W2-3D7) or mutated forms of 3D7 AMA1 (W2-3D7 *Pv*Loop1b + 1E and W2-3D7 *Pv*Loop1E) was calculated by flow cytometry over a single invasion cycle (*n* = 5 wells per line) [45]. Briefly, synchronised ring stage parasites were adjusted to 0.5% parasitaemia and 4% haematocrit and aliquoted into three replicate 96-well plates termed Cycle 0, Cycle 1 Static and Cycle 1 Suspension (*n* = 5 100 μL wells per plate, per parasite line). Cycle 0 (*C* = 0) parasites were allowed to develop into trophozoites for 12 h at 37 °C. Cycle 1 (*C* = 1) parasites were allowed to develop through a single cycle of erythrocyte invasion for 60 h at 37 °C under either static culture conditions or with gentle agitation to maintain erythrocytes in suspension. *C* = 0, *C* = 1 Static and *C* = 1 Suspension trophozoite-stage parasites were fixed for 1 h at room temperature by the addition of glutaraldehyde (ProSciTech) to a final concentration of 0.25% (v/v). After fixation, parasites were washed in HTPBS, stained with 10 × SYBR Green dye (Invitrogen) and 5 × 10^5^ erythrocytes counted per well using a BD FACSCantoII flow cytometer. FACS counts were analysed using FlowJo™ (V6.4.7) software (Treestar). Parasite multiplication was calculated by dividing *C* = 1 parasitaemia by *C* = 0 parasitaemia for each parasite line.

### Statistics

For continuous data, *p*-values were determined for comparisons between two groups using Wilcoxon’s rank test, or Wilcoxon’s signed-rank test for paired data. Analyses were conducted using GraphPad Prism.

## Results

### Divergence in the RON2 sequence of *P. falciparum* from *P. vivax* and *P. knowlesi*

**Fig. 1:**
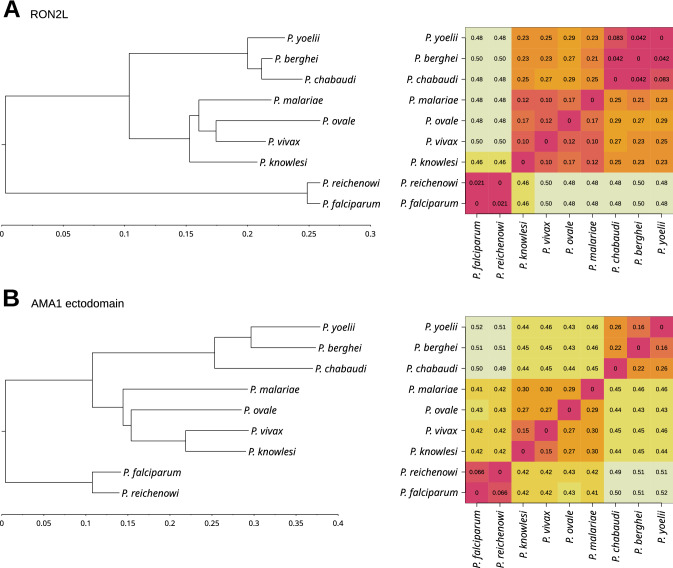
Similarities and differences in RON2 and AMA1 amino acid sequences between *Plasmodium* spp. Neighbour-joining dendrograms (left) showing similarities between the amino acid sequences of *Plasmodium* spp. For RON2L (**A**) and the AMA1 ectodomain (**B**). In both cases, species group as expected by subgenera; the rodent *Vinckeia* species group together, as do species of the *Plasmodium* subgenus (including *P. vivax* and *P. knowlesi*), and the *Laverania* subgenus (*P. falciparum* and *P. reichenowi*). The distance matrices (right) also support this grouping. Notably, sequences from *P. vivax* and *P. knowlesi* are separated by a very low distance, with 0.10 and 0.15 for RON2L and the AMA1 ectodomain, respectively

To understand divergence of RON2 and AMA1 between species, we analysed sequence similarity between *P. falciparum, P. vivax and P. knowlesi*, as well as other representative *Plasmodium* spp. from the *Laverania* and *Vinckeia* subgenera (Fig. 1; Supplementary Figures S1 and S2). Neighbour-joining dendrograms for the RON2-loop sequence and AMA1 ectodomain show that *P. vivax and P. knowlesi* group together, whereas *P. falciparum* grouped separately with *P. reichenowi* (Fig. 1). Distance matrices support this grouping. Notably, sequences from *P. vivax* and *P. knowlesi* are separated by a very low distance (0.10 and 0.15 for RON2-loop and AMA1, respectively), whereas the distance between *P. falciparum* and *P. vivax* or *P. knowlesi* was substantially higher (0.46–0.5 for RON2-loop and 0.42 for AMA1).

### Conservation of AMA1–RON2 binding between *P. vivax* and *P. knowlesi*, but divergence from *P. falciparum*

**Fig. 2:**
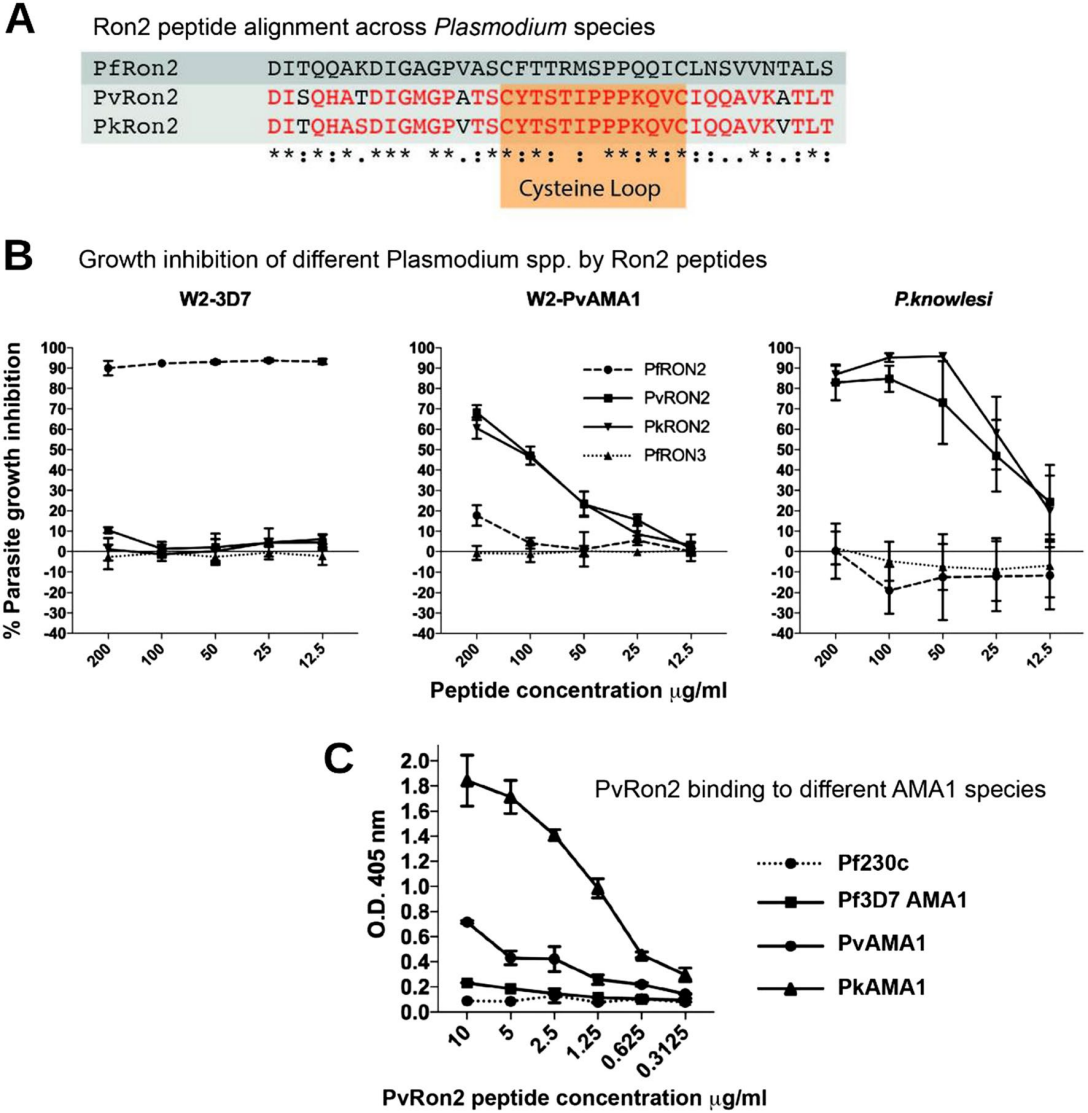
Conserved and divergent functions of RON2-loop among *P. falciparum, P. vivax,* and *P. knowlesi*. **A** Alignment of the RON2L peptide sequences from *P. falciparum* (Pf), *P. vivax* (Pv) and *P. knowlesi* (Pk) orthologues. Amino acids conserved between *Pv*RON2 and *Pk*RON2 are in red. Amino acids that form the disulfide-bonded β-hairpin loop are shaded in orange **B** Invasion inhibition of transgenic *P. falciparum* expressing *Pf*AMA1 (W2-3D7) or *Pv*AMA1 (W2-*Pv*AMA1) or *P. knowlesi* (strain H) by *Pf*RON2, *Pv*RON2, *Pk*RON2 and the control *Pf*RON3 peptides. **C** Binding of *Pv*RON2L peptide to recombinant *Pf*AMA1, *Pv*AMA1, *Pk*AMA1 or Pfs230 (negative control). Data from 2 or more experiments in duplicate and show mean ± SD

We investigated whether there is conservation or species specificity in the RON2-loop for binding to AMA1. A variety of *Pf*RON2 peptides have previously been described. The *Pf*RON2L peptide of 47 amino acids [29] spans Asp-2021 to Lys-2067. The *Pf*RON2sp1 peptide of 39 amino acids, originally reported by [23], spans Asp-2021 to Ser-2059. A peptide of 39 amino acids, also called *Pf*RON2sp1, was subsequently described by [22] and spans Met-2020 to Leu-2058. The sequences of the *Pf*RON2, *Pv*RON2 and *Pk*RON2 C-terminal regions have in common two centrally located cysteine residues that enable the formation of the disulfide-bonded β-hairpin loop structure [23] (Fig. 2A). In *Pf*RON2, Arg-2041 located at the tip of the β-hairpin loop forms a critical interaction with the hydrophobic cleft of *Pf*AMA1. In *Pv*RON2 and *Pk*RON2, the corresponding amino acid is substituted for Thr-2055 [22].

In this study we used two sets of RON2 peptides: (1) three peptides of 39 amino acids: Asp-2021 to Ser-2059 of *Pf*RON2, Asp-2050 to Thr2088 of *Pv*RON2 and Asp-1975 to Thr-2013 of *Pk*RON2 (these are based on the *Pf*RON2sp1 peptide originally described by [22]) and are termed RON2 peptides in this study. (2) two peptides of 48 amino acids: Met-2020 to Lys-2067 of *Pf*RON2L and Met-2049 to Lys-2096 of *Pv*RON2L. These are based on the *Pf*RON2L peptide described by [29] (Asp-2021 to Lys-2067) but with the addition of an N-terminal Met-2020, and are termed RON2L peptides in this study. The shorter RON2 peptides were generally used as inhibitors in assays and had good solubility in aqueous solutions. The longer peptides based on the *Pf*RON2L peptide were used to dissect the functional roles of the N- and C-terminal amino acids flanking the β-hairpin loop core; they had lower solubility in aqueous solutions and needed to be solubilised in 10% DMSO.

The short RON2 peptides of *P. falciparum*, *P. vivax* and *P. knowlesi* (Fig. 2A) were tested for inhibition of invasion against established transgenic *P. falciparum* parasites expressing either *P. falciparum* AMA1 3D7 strain (denoted W2-3D7) and *P. vivax* AMA1 Palo Alto strain (denoted W2-*Pv*AMA1) [31], and against wild-type *P. knowlesi* strain H. The W2-*Pv*AMA1 parasites retain the endogenous *Pf*RON2 protein and have an invasion rate and kinetics that did not differ from *P. falciparum* parasites expressing *Pf*AMA1 [31]. As a negative control peptide, we used a *Pf*RON3 cyclised peptide (Glu-574 to Lys-603).

*Pf*RON2 peptide strongly inhibited the invasion of transgenic W2-3D7 parasites expressing *Pf*AMA1 parasites (Fig. 2B). In contrast, *Pv*RON2 and *Pk*RON2 peptides had little or no inhibition of parasites expressing *Pf*AMA1. *Pv*RON2 and *Pk*RON2 peptides both effectively inhibited parasites expressing *Pv*AMA1 (W2-*Pv*AMA1 parasites), whereas *Pf*RON2 showed only minor inhibition at the highest concentration tested (200 µg/mL), but no inhibition at lower concentrations. Similarly, both *Pv*RON2 and *Pk*RON2 peptides effectively inhibited *P. knowlesi* parasites, whereas *Pf*RON2 gave no significant inhibition. No inhibition was observed for the *Pf*RON3 control peptide when tested against any of the parasite strains, supporting specificity of the assays (Fig. 2B). These findings led us to conclude that while the pairing of *Pf*AMA1 with *Pf*RON2 is highly species-specific, there is a level of functional conservation in the AMA1–RON2 pairing between *P. vivax* and *P. knowlesi*.

Direct binding of *Pv*RON2L peptide to recombinant AMA1 corresponding to the three species was studied to provide supporting evidence for conclusions made from invasion inhibition studies (ectodomains of *Pf*AMA1 (3D7 allele), *Pv*AMA1 (Palo Alto allele) and *Pk*AMA1 (strain H allele) were expressed; Supplementary figure S3). When tested in a plate-based binding assay, *Pv*RON2L demonstrated high binding to *Pv*AMA1 and higher binding to *Pk*AMA1 (Fig. 2C). However, there was little binding to *Pf*AMA1, and no binding to the Pfs230 negative control protein. This pattern of binding is consistent with invasion inhibition data indicating cross-inhibition of *P. vivax* and *P. knowlesi* by *Pv*RON2 and *Pk*RON2 peptides, but lack of inhibition of *P. falciparum* by these same peptides (Fig. 2B).

### Sequences flanking the β-hairpin loop of RON2L are not required for species-specific AMA1 binding

To evaluate whether regions adjacent to the RON2 β-hairpin loop influence AMA1 binding, we tested the longer *Pf*RON2L (Met-2020 to Lys-2067) and *Pv*RON2L (Met-2049 to Lys-2096) peptides. Additionally, we synthesised hybrid RON2L peptides in which the N- and C-terminal amino acids (either side of the β-hairpin loop) were replaced with those from *P. vivax*. The hybrid peptide *PvPf*RON2L contains the central β-hairpin loop of *Pf*RON2, flanked by the N- and C-terminal amino acids of *Pv*RON2L. The *PfPv*RON2L hybrid peptide contained the central β-hairpin loop of *Pv*RON2, flanked by the N- and C-terminal amino acids of *Pf*RON2L. These peptides required solubilisation in 10% DMSO; therefore, relative inhibitory activity of peptides was measured against DMSO controls of the same concentration.

**Fig. 3:**
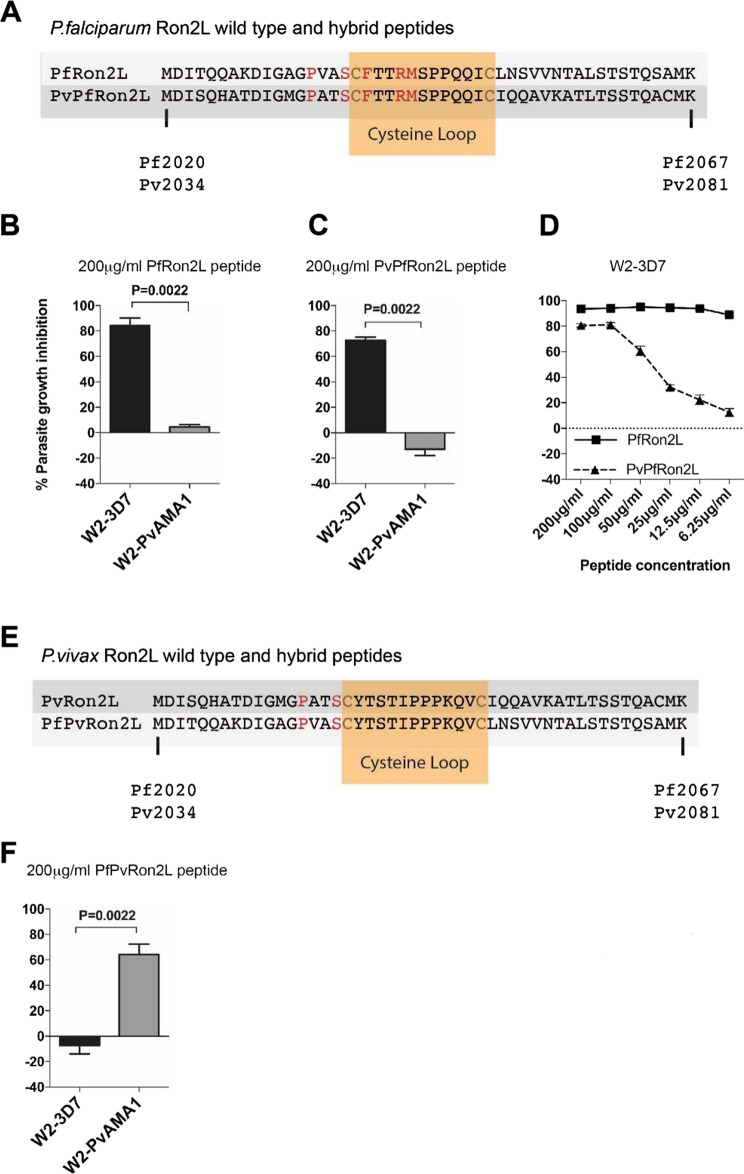
The central β-hairpin loop of RON2L peptides mediate species-specific inhibition of invasion. **A** Alignment of *P. falciparum* (*Pf*) RON2L and the chimeric *P. vivax* flank/*P. falciparum* central β-hairpin loop hybrid RON2L (*PvPf*RON2L) peptides. Amino acids that differ between *Pf*RON2L and *Pv*RON2L are in red. Amino acids that form the β-hairpin loop are shaded in orange. **B** Invasion inhibition of *P. falciparum* expressing *Pf*AMA1 (W2-3D7) or *Pv*AMA1 (W2-*Pv*AMA1) by 200 μg/mL *Pf*RON2L peptides. **C** Inhibition of transgenic *P. falciparum* expressing *Pf*AMA1 (W2-3D7) or *Pv*AMA1 (W2-*Pv*AMA1) by 200 μg/mL *PvPf*RON2L. **D** Inhibition of transgenic *P. falciparum* expressing *Pf*AMA1 (W2-3D7) by *Pf*RON2L and *PvPf*RON2L peptides. **E** Alignment of *Pv*RON2L (*Pv*RON2L) and the chimeric *P. falciparum* flank/*P. vivax* central β-hairpin loop hybrid RON2L (*PfPv*RON2L) peptides. Conserved amino acids are in red. Amino acids that form the β-hairpin loop are shaded in orange. **F** Inhibition of transgenic *P. falciparum* expressing *Pf*AMA1 (W2-3D7) or *Pv*AMA1 (W2-*Pv*AMA1) by 200 μg/mL *PfPv*RON2L

At 200 μg/mL, both the longer *Pf*RON2L and hybrid *PvPf*RON2L (sequences shown in Fig. 3A) effectively inhibited the invasion of *P. falciparum* expressing *Pf*AMA1 (W2-3D7). In contrast there was little inhibition of parasites expressing *Pv*AMA1 (Fig. 3B,C). Titrating the *Pf*RON2L and hybrid *PvPf*RON2L peptides against parasites expressing *Pf*AMA1 (W2-3D7) showed that the *PvPf*RON2L hybrid peptide had lower inhibitory activity compared to the *Pf*RON2L peptide (Fig. 3D). Therefore, the central β-hairpin loop appears to mediate species-specific AMA1 binding by *Pf*RON2L peptides, and the N- and C-terminal amino acids appear to play a role in enhancing binding to AMA1. This is consistent with a previous study reporting that a longer *Pf*RON2L peptide (amino acids 2021–2059) had higher affinity binding to recombinant *Pf*AMA1 than a shorter *Pf*RON2 peptide (amino acids 2027–2055) [23, 29]. Crystal structures of AMA1-RON2L complexes identified amino acids either side of the central β-hairpin loop that make contact with AMA1 and these amino acids differ between *Pf*RON2L and *Pv*RON2L [22]. Therefore, these additional contact points would be absent with the *PvPf*RON2L peptide interaction with *Pf*AMA1 and likely explains their lower inhibitory effect against parasites expressing *Pf*AMA1. The longer *Pv*RON2L peptide (including the additional N- and C-terminal amino acids) was poorly soluble, even in DMSO. However, the hybrid *PfPv*RON2L peptide (Fig. 3E) could be evaluated and was found to strongly inhibit invasion of parasites expressing *Pv*AMA1 (W2-*Pv*AMA1), but did not inhibit invasion of *P. falciparum* expressing *Pf*AMA1 (W2-3D7) (Fig. 3F). This further supports the importance of the central β-hairpin loop for defining species-specific AMA1-binding activity.

### Identification of key amino acids in *Pv*AMA1 Loop1E for species-specific RON2 binding

**Fig. 4:**
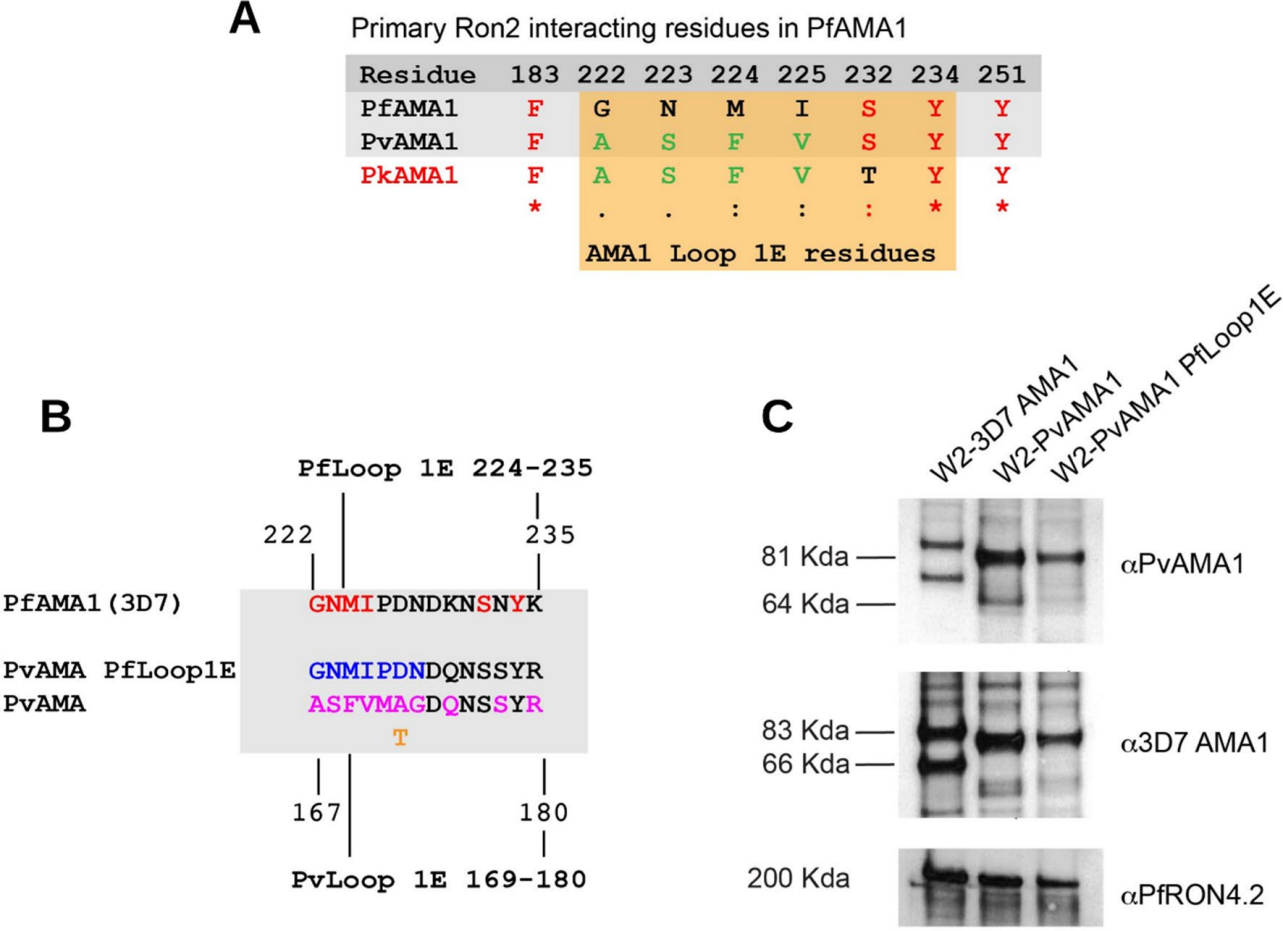
Differences between *Plasmodium* species in AMA1 amino acids that interact with RON2-loop, and generation of a hybrid *Pv*AMA1-*Pf*Loop1E mutant parasite line. **A** Key *P. falciparum* (*Pf*) AMA1 amino acid residues that have been identified to play a role in binding to *Pf*RON2 aligned against those found at the same relative position in *Pv*AMA1 and *Pk*AMA1 (residue numbers are *Pf*AMA1 specific). Amino acids conserved across all three species are in red; except amino acid 232, which is the same in *Pf*AMA1 and *Pv*AMA1, but differs in *Pk*AMA1. Amino acids conserved between *Pv*AMA1 and *Pk*AMA1 only are in green. Amino acids located in the Loop1E domain (222–234) are shaded in orange. **B** Alignment of *Pf*AMA1 and *Pv*AMA1 Loop1E amino acid sequences. Proposed *Pf*RON2 interacting residues are in red. *Pv*AMA1 (Palo Alto) residues that differ from *Pf*AMA1 (3D7) are shown in magenta and an alternative amino acid present in other *Pv*AMA1 alleles is shown in orange. The hybrid *Pv*AMA1 incorporating *Pf*AMA1 Loop1E is shown, with *Pf*AMA1-specific amino acid residues in blue. **C** Transgenic *P. falciparum* expressing a chimeric *Pv*AMA1 with the *Pf*AMA1-Loop1E sequence (denoted *Pv*AMA1*Pf*Loop1E) was generated (see Supplementary Material, Figure S5). Analysis of *Pv*AMA1*Pf*Loop1E protein expression was investigated by western blot. Schizont protein extracts were prepared from transgenic *P. falciparum* lines expressing either *Pf*AMA1 (W2-3D7), *Pv*AMA1 (W2-*Pv*AMA1) or *Pv*AMA1*Pf*Loop1E (W2-*Pv*AMA1*Pf*Loop1E) and run in non-reducing conditions (3–8% acrylamide gradient gel). Western blots were probed with anti-3D7 *Pf*AMA1 (3D7 allele) or anti-*Pv*AMA1 polyclonal antibodies (20 μg/mL IgG) raised in rabbits. Antibodies labelled the expected ~ 83 kDa and ~ 66 kDa species of *Pf*AMA1 protein, whereas a smaller ~ 81 kDa band is seen in transgenic parasites expressing *Pv*AMA1 and *Pv*AMA1*Pf*Loop1E mutant proteins. Equal loading of schizont samples was confirmed by probing blots with anti-*Pf*RON4.2 mAb (5 μg/mL IgG)

To further understand the function of AMA1–RON2 binding in erythrocyte invasion, we mutated key RON2-binding residues in *Pv*AMA1 expressed in our transgenic *P. falciparum* model. Previous studies suggest that the *Pf*RON2L peptide interacts with eight key amino acids of *Pf*AMA1 [23]. Of these, amino acids Phe-183 and Tyr-251 are conserved across *Pf*AMA1, *Pv*AMA1 and *Pk*AMA1 (Fig. 4A). The other six are located adjacent to, or form part of, the N-terminal face of Loop1E of *Pf*AMA1. *Pv*AMA1 differs from *Pf*AMA1 at four out of six amino acids in Loop1E, whereas *Pk*AMA1 differs from *Pv*AMA1 for only one of the six amino acids. A high level of sequence conservation across these six amino acids of AMA1 Loop1E is observed between *P. vivax*, *P. ovale, P. malariae*, *P. knowlesi*, *P. coatneyi* and *P. cynomolgi* (Supplementary figure S4). We mutated the Loop1E sequence of *Pv*AMA1, replacing it with the sequence from *Pf*AMA1 (3D7 allele). To be conservative, we did not mutate the entire *Pv*AMA1-Loop1E sequence; rather only seven amino acids in the N-terminal face were replaced (equivalent to Gly-222 to Asn-228 in *Pf*AMA1)(Fig. 4B). While Ser-232 and Tyr-234 of *Pf*AMA1 have also been reported to form part of the binding pocket, they are conserved between *Pv*AMA1 and *Pf*AMA1 and so were not changed in *Pv*AMA1.

*P. falciparum* parasites (W2mef strain) were transfected with a plasmid containing the *Pv*AMA1*Pf*Loop1E gene (Figure S5A). Complete allelic exchange in the cloned parasite line (W2-3D7 *Pv*Loop1E) was confirmed by PCR (Figure S5B). Western blots of schizont protein extracts probed with anti-AMA1 antibodies detected the expected *Pf*AMA1 bands at ~ 83 kDa and ~ 66 kDa processed bands (Fig. 4C), that have been previously described [46]. In contrast, western blots of transgenic *P. falciparum* W2-*Pv*AMA1 or W2-*Pv*AMA1*Pf*Loop1E schizont protein extracts detected a band with lower molecular weight (predicted to be ~ 81 kDa) and a less abundant band at ~ 63 kDa band, similar to previous findings of labelling *Pv*AMA1 in western blots [31]. The size shift supports the analysis by PCR indicating that replacement of *Pf*AMA1 with *Pv*AMA1 genes was successful (Figure S5B), and is consistent with the predicted molecular weights of the *Pv*AMA1 [31] and *Pv*AMA1*Pf*Loop1E proteins and confirmed the loss of endogenous *Pf*AMA1 protein expression in W2-*Pv*AMA1*Pf*Loop1E parasites (Fig. 4C).

**Fig. 5:**
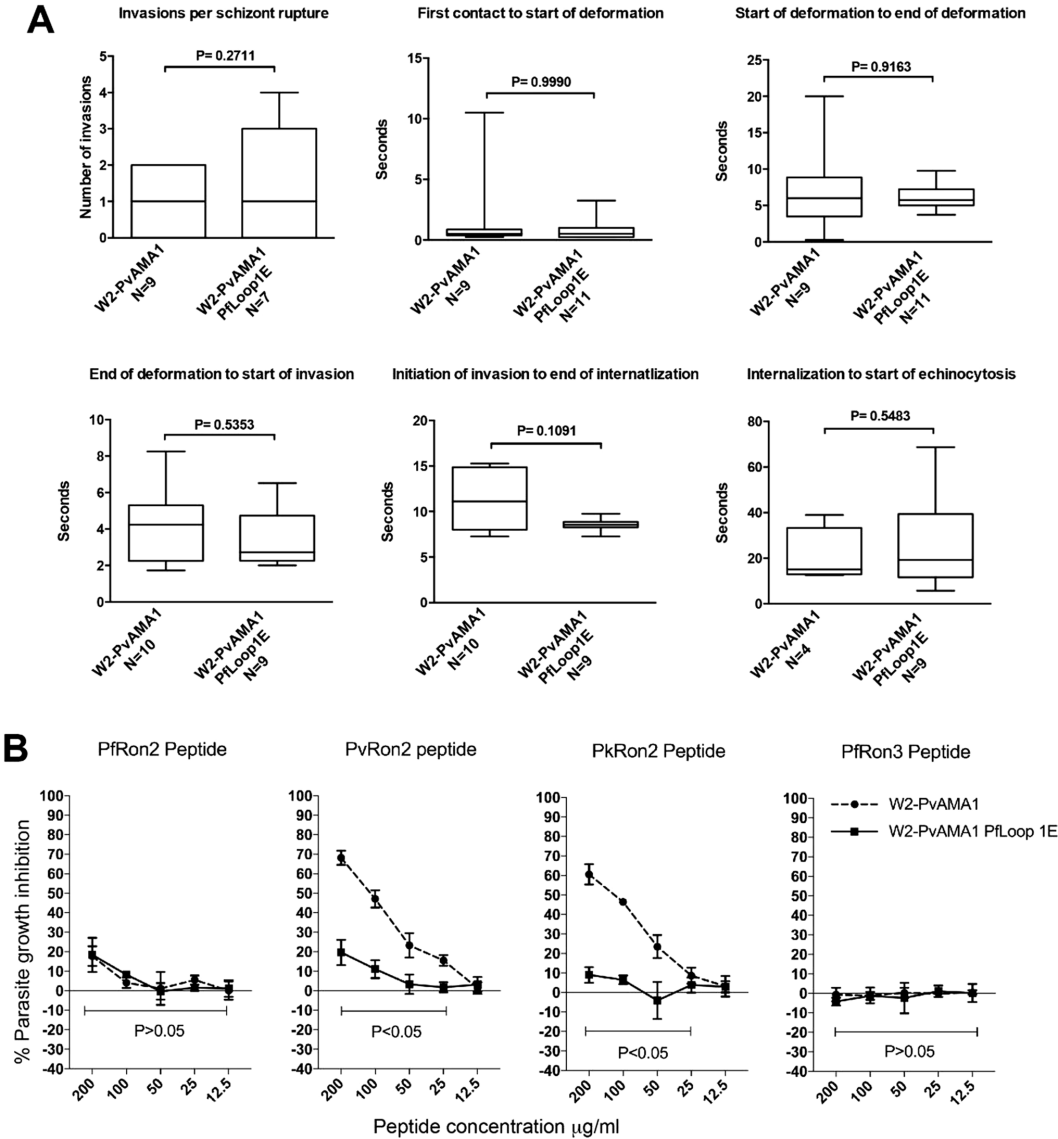
Parasites expressing *Pv*AMA1 incorporating *Pf*AMA1-Loop1E amino acids invade erythrocytes normally but display reduced inhibition by *Pv*RON2 peptide. **A** Merozoite invasion kinetics of transgenic *P. falciparum* (*Pf*) lines expressing *P. vivax* (*Pv*) AMA1 (W2-*Pv*AMA1) or *Pv*AMA1*Pf*Loop1E (W2-*Pv*AMA1*Pf*Loop1E) as measured by live-cell imaging at six consecutive stages of erythrocyte invasion. *N* = number of events filmed per stage of invasion. Statistical analysis by unpaired *t* test (invasions per schizont rupture) or Mann–Whitney *U* test (all other panels). The bar graphs are box-and-whisker type, showing the median (line), inter-quartile range (box) and range (whiskers). **B** Differential inhibition of transgenic W2-*Pv*AMA1 and W2-*Pv*AMA1*Pf*Loop1E parasite lines by titrated *Pf*RON2, *Pv*RON2, *Pk*RON2 or the negative control *Pf*RON3 peptide. Mean and standard deviation of two assays performed in duplicate wells. Statistical analysis by paired *t* test for each individual peptide concentration. *P*-value score for all peptide concentrations ranges defined by the lower bar

Analysis by live microscopy showed that merozoites of the W2-*Pv*AMA1*Pf*Loop1E parasites invaded erythrocytes in vitro with similar kinetics to *Pv*AMA1 parasites, suggesting the functions of *Pv*AMA1 and *Pv*AMA1*Pf*Loop1E are biologically equivalent. There was no significant difference between the parasite lines for all parameters examined, including the time of first merozoite–erythrocyte contact to the start and end of deformation, or initiation and completion of invasion (Fig. 5A). Mutation of Loop1E of *Pv*AMA1 greatly reduced the ability of *Pv*RON2 peptide or *Pk*RON2 peptide to inhibit invasion compared to *P. falciparum* expressing unmodified *Pv*AMA1 (Fig. 5B), suggesting binding of *Pv*RON2 and *Pk*RON2 to *Pv*AMA1 is largely ablated by mutation of Loop1E. Interestingly, there was no significant gain in inhibition by the *Pf*RON2 peptide (Fig. 5B). This suggests the cluster of six amino acids in *Pv*AMA1 Loop1E plays a major role in determining species-specific binding to *Pv*RON2 and *Pk*RON2. These findings, in which RON2-loop-binding activity of *Pv*AMA1 has been greatly reduced, suggest that the binding of *Pv*AMA1 to the RON2-loop is not essential for invasion. The ability of *Pv*AMA1 to complement the function of *Pf*AMA1 in *P. falciparum* invasion is not explained by an ability of *Pv*AMA1 to bind *Pf*RON2.

### Mutations in Loop1E of *Pf*AMA1 reduce *Pf*RON2 binding, but do not affect parasite invasion

**Fig. 6:**
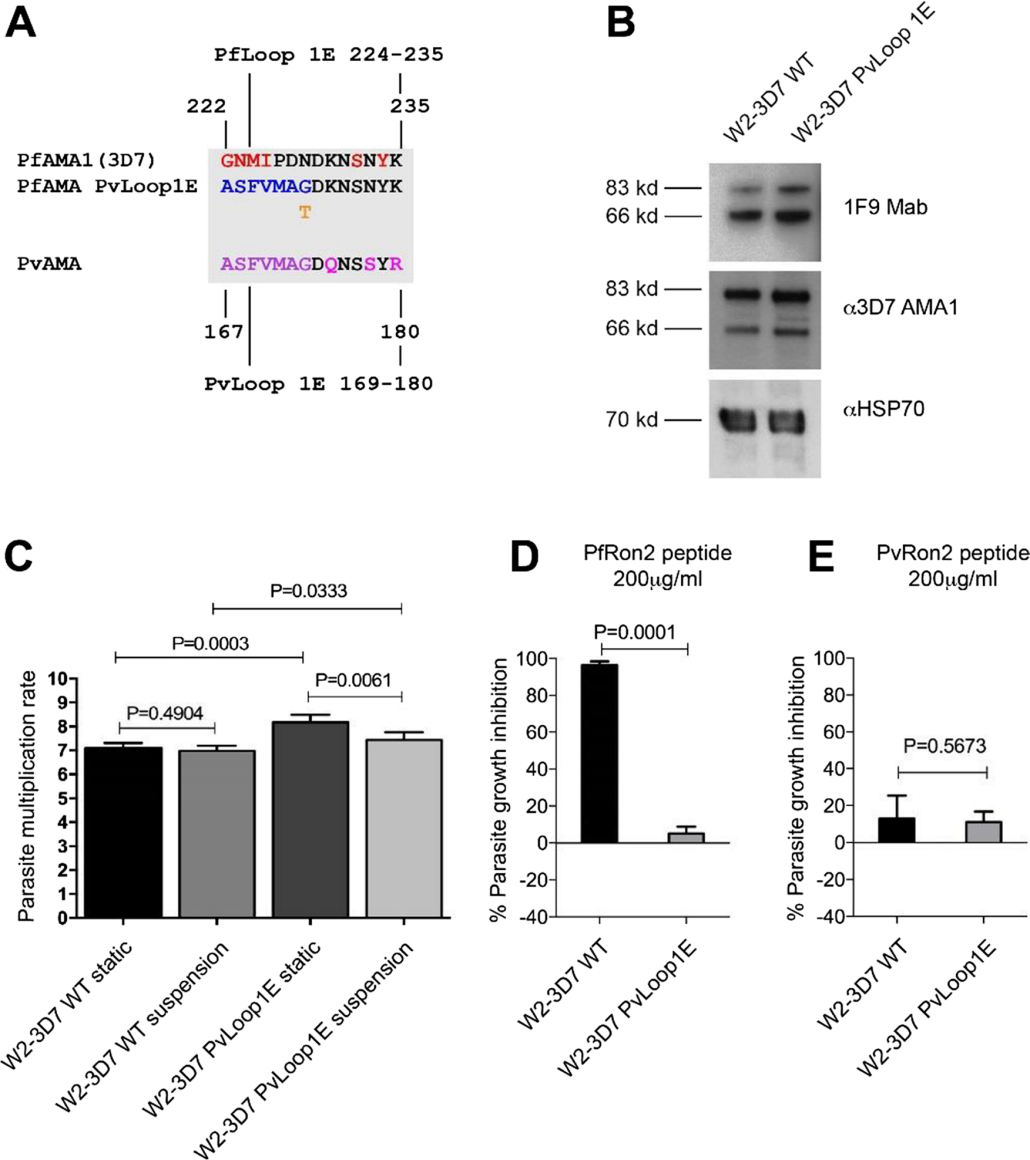
Parasites expressing *Pf*AMA1 incorporating *Pv*AMA1-Loop1E amino acids replicate in erythrocytes normally but display reduced invasion inhibition by *Pf*RON2 peptide. **A** Alignment of *Pf*AMA1 and *Pv*AMA1 Loop1E amino acid sequences. *Pf*RON2 interacting residues are in red. *Pv*AMA1 (3D7 strain) residues that differ from *Pf*AMA1 are in magenta, and an alternative amino acid used by other *Pv*AMA1 alleles is in orange. The hybrid *Pf*AMA1 incorporating *Pv*AMA1-Loop1E is shown, with *Pv*AMA1-specific amino acid residues in blue. **B** Western blots of schizont material expressing *Pf*AMA1 (W2-3D7) or *Pf*AMA1 with *Pv*Loop1E (W2-3D7 *Pv*Loop1E) probed with 2.5 μg/mL 1F9 mAb, 20 μg/mL polyclonal rabbit anti-3D7 AMA1 IgG or 1:500 diluted polyclonal rabbit anti-HSP70 antisera. **C** *P. falciparum* multiplication rates were measured for W2-3D7 and W2-3D7 *Pv*Loop1E transgenic *P. falciparum*. Rates were calculated for parasite replication in static erythrocyte culture and suspended erythrocyte culture conditions. Mean and standard deviation of each condition was tested over five wells. Statistical analysis was done by paired *t* test. **D, E** Differential inhibition of parasites expressing *Pf*AMA1 and *Pf*AMA1-*Pv*Loop1E by 200 μg/mL *Pf*RON2 peptide (**D**) or 200 μg/mL *Pv*RON2 peptide (**E**). Mean and standard deviation of two assays was performed in triplicate wells. Statistical analysis was by paired *t* test

To further understand the interaction between *Pf*AMA1 and RON2-loop, we generated parasites with mutation of key amino acids in *Pf*AMA1. Seven amino acids of the N-terminal face of Loop1E of *Pf*AMA1 (Gly-222 to Asn-228) were substituted with those from *Pv*AMA1 (Fig. 6A). This was the reciprocal of the mutations generated in *Pv*AMA1-*Pf*Loop1E parasites (Fig. 4B). Parental W2mef *P. falciparum* was transfected with the plasmid containing the *Pf*AMA1*Pv*Loop1E gene (Figure S6A). Complete allelic exchange in the cloned parasite line, W2-3D7-*Pv*Loop1E, was confirmed by PCR (Figure S6B). Expression of the *Pf*AMA1*Pv*Loop1E mutant protein in transgenic *P. falciparum* was confirmed by probing schizont protein extracts with polyclonal antibodies and a mAb to *Pf*AMA1 (Fig. 6B).

The insertion of *Pv*Loop1E amino acids into *Pf*AMA1 had no detrimental effect on *P. falciparum* invasion and growth in vitro. In fact, the multiplication rate of *Pf*AMA1*Pv*Loop1E parasites was slightly higher than that of the control *Pf*AMA1 parasites (15% and 6% greater under static and suspension culture conditions, respectively) (Fig. 6C). Mutation of the amino acids in Loop1E of *Pf*AMA1 greatly reduced the ability of *Pf*RON2 peptide to inhibit invasion (Fig. 6D). However, the insertion of *Pv*AMA1 Loop1E amino acids did not lead to any significant gain in inhibition by *Pv*RON2 peptide (Fig. 6E). This is consistent with results obtained for the *Pv*AMA1 Loop1E mutant line and supports the concept that species-specific binding of the RON2L peptide to AMA1 is mediated by Loop1E, and the RON2L–AMA1 interaction is not essential for invasion.

### Changes in invasion-inhibitory activity of monoclonal antibodies against *Pf*AMA1 following mutation of Loop1E

**Fig. 7:**
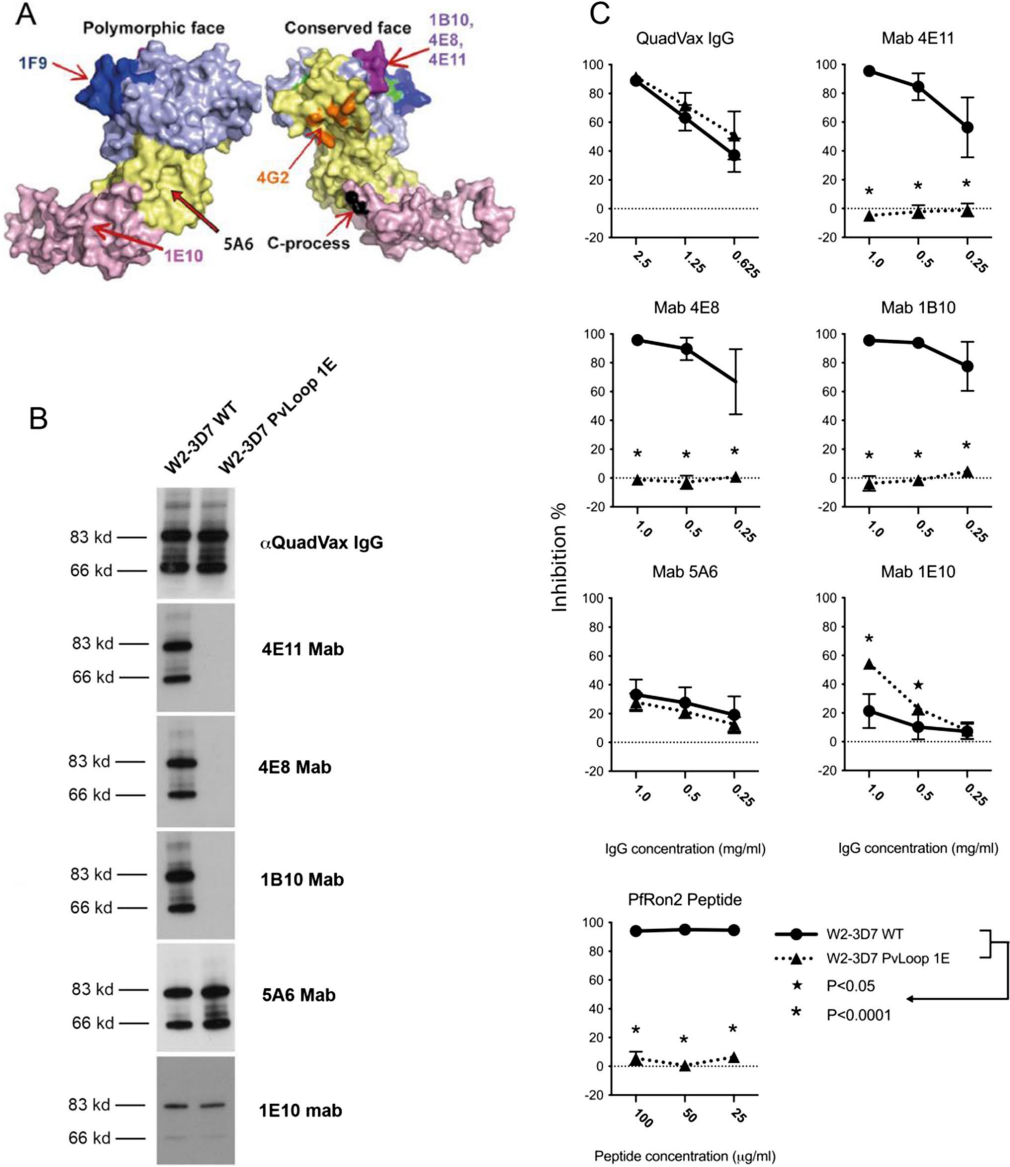
Inhibitory activity of monoclonal antibodies against *Pf*AMA1 following mutation of Loop1E. **A** 3D structure of AMA1 [41] with arrows indicating the approximate binding sites of mAbs used in this study (1B10, 4E8, 4E11, 1E10, and 5A6). Other known inhibitory mAbs 1F9 and 4G2 are shown for reference. **B** Western blots of schizont material from *P. falciparum* expressing wild-type *Pf*AMA1 (W2-3D7) or *Pv*AMA1 with *Pv*Loop1E (W2-3D7 *Pv*Loop1E) were probed with the Loop1E binding mAbs 4E11, 4E8, 1B10 or the domain 2 binding mAb 5A6 as a loading control. All antibodies were tested at a final concentration of 2.5 µg/mL IgG. **C** Differential growth inhibition of transgenic W2-3D7 and W2-3D7 *Pv*Loop1E parasite lines by mAbs 4E11, 4E8, 1B10, 5A6, and 1E10 or by *Pf*RON2 peptide. The *Y*-axis shows % inhibition; mean and standard deviation of two inhibition assays performed in triplicate wells. Statistical analysis was by paired *t* test; *indicates *p* < 0.05

We investigated the invasion-inhibitory activity of mAbs to *Pf*AMA1, including mAbs that have been previously reported to bind *Pf*AMA1-Loop1E (4E11, 4E8 and 1B10) [41], and mAbs to domains 2 and 3 (5A6 and 1E10, respectively) (Fig. 7). The Loop1E mAbs were previously shown to block *Pf*RON2 peptide binding to *Pf*AMA1 [41]. In western blots with schizont protein extracts, Loop1E mAbs labelled *Pf*AMA1 in parasites expressing *Pf*AMA1, but there was a complete loss of reactivity against parasites expressing the *Pf*AMA1*Pv*Loop1E mutant, confirming that these mAbs target Loop1E and that changes in the seven amino acid stretch that differs between *Pf*AMA1 and *Pv*AMA1 is important for mAb epitope recognition. In contrast, polyclonal rabbit IgG raised against *Pf*AMA1 (QuadVax [41]), and mAb 5A6 (domain 2) and mAb 1E10 (domain 3) labelled both AMA1 types. Loop1E binding mAbs significantly inhibited parasites expressing *Pf*AMA1, but did not inhibit *Pf*AMA1*Pv*Loop1E parasites. This confirms that targeted mutation has disrupted the function of *Pf*AMA1 Loop1E, complementing earlier findings evaluating inhibition by RON2 peptides (Fig. 6), and demonstrates that these mutations allow parasites to escape antibody inhibition. mAb 5A6, targeting domain 2, inhibited *Pf*AMA1 and *Pf*AMA1 Loop1E mutant parasite lines at similar levels. Interestingly, mAb 1E10, targeting domain 3, showed greater inhibition of *Pf*AMA1*Pv*Loop1E parasites, suggesting that disruption of AMA1-Loop1E binding to the RON2-loop leads to a greater reliance on domain 3 interactions for invasion.

## Discussion

**Fig. 8:**
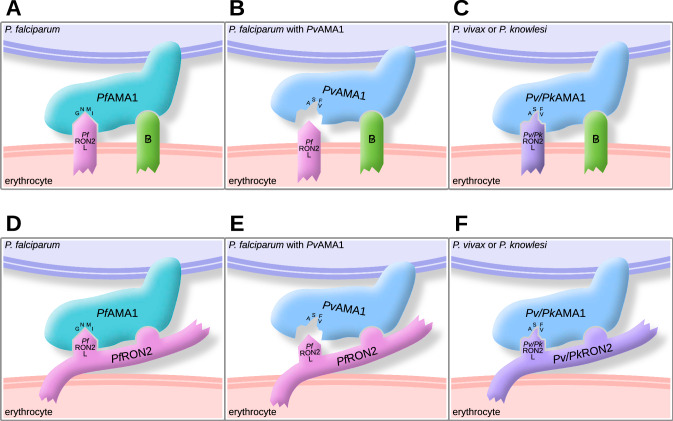
Models of possible AMA1 binding interactions for invasion. AMA1 is present on the merozoite surface and the RON2-loop (labelled as RON2L) is present on the erythrocyte surface. AMA1 is known to bind to the RON2-loop and new data suggest that there is an additional binding interaction of AMA1. In one model (panels **A–C**), AMA1 binds to Receptor B, which is a proposed molecule on the erythrocyte surface, yet to be identified, that acts a second receptor for AMA1 binding. In the alternative model (panels **D–E**), the second binding interaction of AMA1 is to a second site on RON2 that is separate to the RON2-loop. **A, D**. In *P. falciparum* parasites, *Pf*AMA1 binds to the *Pf*RON2-loop (*Pf*RON2L) and either Receptor B (**A**) or another region of *Pf*RON2 (**D**) to mediate invasion. Key amino acids of *Pf*AMA1 Loop1E for binding to RON2 are indicated (G, N, M, and I), as identified in this study. **B, E**. In genetically modified *P. falciparum* parasites expressing *Pv*AMA1, *Pv*AMA1 does not bind to the *Pf*RON2-loop, but binds to either Receptor B (**B**) or another region of *Pf*RON2 (**E**), allowing invasion to occur. The lack of binding of *Pv*AMA1 to the *Pf*RON2-loop is due to differences in key amino acids of AMA1 Loop1E between *P. vivax* and *P. falciparum* (ASFV for *Pv*AMA1 and GNMI for *Pf*AMA1). **C, F**. In *P. vivax* and *P. knowlesi* parasites, *Pv*AMA1 and *Pk*AMA1 can bind to the *Pv*RON2-loop or *Pk*RON2-loop, as well as Receptor B (**C**) or another region on *Pv*RON2 or *Pk*RON2 (**F**). There is conservation of AMA1-RON2-loop binding between the two species. The amino acid sequences of the β-hairpin loop of RON2 are identical between *P. vivax* and *P. knowlesi*. Of the key RON2-interacting amino acids of AMA1 Loop1E, only one amino acid differs between *P. vivax* and *P. knowlesi*. The native conformation and surface orientation of RON2 is not currently fully defined

In this study, we investigated interactions between AMA1 and RON2 for erythrocyte invasion by *P. falciparum*, *P. vivax* and *P. knowlesi* to gain new insights to inform the development of novel vaccines and therapeutics against the three species causing malaria. Our findings support several important conclusions, based on studies with different RON2-loop peptides as inhibitors and parasites expressing *Pf*AMA1, *Pv*AMA1 or *Pk*AMA1; mutation studies of key AMA1 residues that bind RON2-binding residues in AMA1; and antibody inhibition assays. A major novel finding was that high-affinity binding of RON2-loop to AMA1 is not essential for invasion, which changes the current model of invasion interactions for AMA1 (Fig. 8). Furthermore, AMA1 can tolerate mutations that enable evasion of inhibitory antibodies or molecules targeting the AMA1–RON2 binding interaction, without impacting on erythrocyte invasion. Together, these findings indicate that targeting of AMA1 with vaccines and therapeutics will need to be broader than just the interaction of AMA1 with the RON2-loop to maximise efficacy and reduce the risk of vaccine escape. An additional new insight is that there is conservation in AMA1–RON2 binding between *P. vivax* and *P. knowlesi*, whereas *Pf*AMA1 Loop1E and *Pf*RON2 β-hairpin loop have diverged such that there is little capacity for cross-species binding between this species and *P. vivax* or *P. knowlesi*. The minimal β-hairpin loop of RON2 defines species specificity and function, and key residues in Loop1E of AMA1 that are required for binding RON2-loop have diverged between *P. falciparum* and *P. vivax* species to result in species specificity of the AMA1–RON2-loop interaction. In contrast, the more closely-related *P. vivax* and *P. knowlesi* have not substantially diverged, conserving cross-species binding of the RON2-loop to AMA1. These knowledge of specific interactions, conservation and divergence, will be valuable for informing vaccine design for all three malaria species.

Several lines of evidence support the conclusion that AMA–RON2-loop binding is not essential for invasion. First, replacement of *Pf*AMA1 with *Pv*AMA1 in *P. falciparum* parasites did not lead to detectable changes in the invasion rate or kinetics in vitro [31]. *Pf*RON2 is still expressed in these chimeric parasites, yet *Pv*AMA1 has little binding to the *Pf*RON2-loop as indicated by little inhibition of parasites expressing *Pv*AMA1 by *Pf*RON2 peptides (Fig. 2B). Supporting this, prior studies using surface plasmon resonance and recombinant proteins found no detectable binding of *Pf*RON2 to *Pv*AMA1 [22]. Furthermore, mutation of the *Pf*AMA1 Loop1E largely ablated binding to the *Pf*RON2-loop, as indicated by the loss of inhibition by *Pf*RON2 peptides (Fig. 5D). However, invasion still proceeded without any obvious defect in invasion kinetics. Antibodies to *Pv*AMA1 inhibited invasion of parasites expressing *Pv*AMA1, and antibodies to domain 2 and 3 of *Pf*AMA1 could inhibit invasion of the *Pf*AMA1 Loop1E mutant parasites. This indicates that AMA1 still plays an important function in invasion in these modified parasites, suggesting that AMA1 has other important interactions or functions during invasion, aside from binding to the RON2-loop (Fig. 8). It was notable that a mAb targeting domain 3 of *Pf*AMA1 showed greater inhibitory activity against parasites with mutated *Pf*AMA1 Loop1E, which largely ablated RON2-loop binding (Fig. 7). This suggests that during invasion there is a second interaction of AMA1, potentially to a novel receptor on erythrocytes or another region of RON2, which is large protein (2189 amino acids). Currently, the native conformation and surface orientation of RON2 is poorly defined. A prior study reported that recombinant *Pv*AMA1 can bind the surface of erythrocytes, although the magnitude of binding was low and receptors have not been identified [47]. Another study reported that domain 3 of *Pf*AMA1 expressed on the surface of Chinese hamster ovarian cells could bind trypsin-treated erythrocytes [48]. However, full-length AMA1 did not bind erythrocytes, and domain 3 did not bind to untreated erythrocytes, only to trypsin-treated erythrocytes. Therefore, additional binding interactions of AMA1 and the potential role of domain 3 in invasion remains unclear and further research is needed. In addition to protein-binding interactions, evidence suggests that AMA1 has other important roles, and it is possible that a protein binding interaction for AMA1 is not essential for invasion. Prior studies with *P. falciparum* showed that the cytoplasmic tail of *Pf*AMA1 is phosphorylated during invasion and this appears to be important for efficient invasion of erythrocytes [49, 50]. Exposure of *P. falciparum* merozoites to a low potassium concentration, as found in blood plasma, can provide a signal for the release of *Pf*AMA1 (and other proteins) from apical organelles to the merozoite surface after schizont rupture [51]. Given the importance of AMA1 in invasion and its potential as a vaccine and therapeutic target, further research is needed to fully define the functions of AMA1.

Taken together, our results suggest there is redundancy in AMA1–RON2-loop binding; while it is involved in invasion, it is not essential, and invasion can be mediated via additional AMA1 interactions. Our data support a role for AMA1–RON2-loop binding in invasion by *P. vivax* and *P. knowlesi*, even if the interaction is not essential. *Pv* or *Pk*RON2L peptides could inhibit invasion by *P. knowlesi* parasites (Fig. 2B)*.* Surprisingly, *Pv*RON2 peptides could inhibit invasion of parasites expressing *Pv*AMA1 even though the interaction it is not essential for invasion. This might be explained by the binding of exogenous *Pv*RON2 peptides resulting in steric hindrance of other interactions or causing conformational changes in *Pv*AMA1 that impact on other interactions. In *P. falciparum* expressing *Pv*AMA1, it is possible that *Pf*RON2-loop can bind *Pv*AMA1 with low affinity, which may be sufficient for invasion when combined with a second AMA1 binding interaction. When excess *Pv*RON2 peptide is present in invasion assays, high-affinity binding to *Pv*AMA1 may outcompete the low-affinity *Pf*RON2 binding, leading to inhibition of invasion. However, there was little inhibition of invasion of *Pv*AMA1 parasites by *Pf*RON2 peptides, which would be expected if this model was correct, suggesting that there is not significant binding between *Pv*AMA1 and *Pf*RON2.

Our findings have significant implications for the development of vaccines and therapeutics targeting AMA1. First, our findings show that small changes in sequence can mediate escape of inhibitory antibodies and molecules targeting the AMA1–RON2 interaction. Second, our data indicate that the binding of AMA1 to the RON2 β-hairpin loop is not essential for invasion and that other interactions with AMA1 occur during invasion. Therefore, targeting the AMA1-RON2-loop interaction may not be ideal or sufficient to achieve highly efficacious vaccines, or may be at risk of escape, since key amino acid changes can be tolerated. Revealing other molecular interactions and vaccine strategies that additional AMA1 interactions may enable vaccines with higher protective efficacy. To date, vaccines based on *Pf*AMA1 have not shown substantial clinical efficacy or generated potent invasion-inhibitory antibodies in clinical trials [19, 52, 53]. Our data showing greater inhibition by a mAb targeting domain 3 in parasites with mutation of *Pf*AMA1 Loop1E suggest that domain 3 should be a focus of future studies to understand its function and potential to be targeted by vaccines. Further, a prior study found synergistic inhibition of invasion of *P. falciparum* when combining AMA1 antibodies to domain 3 and domain 2 [41]. Parasite lines generated here will be useful tools to progress these concepts in AMA1 vaccine design for the three species, as well as evaluating immune responses in clinical trials.

Our studies demonstrated divergence of AMA1–RON2-loop binding specificity between *P. falciparum* and *P. vivax* using different RON2 peptide inhibitors of invasion and mutation studies of *Pf*AMA1 and *Pv*AMA1 in live parasites. In contrast, AMA1–RON2-loop binding was conserved between *P. vivax* and *P. knowlesi*. Prior crystallography studies of the *Pv*AMA1–*Pv*RON2L homocomplex and *Pf*AMA1–*Pv*RON2L heterocomplex identified amino acids involved in AMA1–RON2 binding interactions [22]. Using invasion assays with live parasites, our studies functionally demonstrated that the disulfide-bonded β-hairpin loop of RON2 mediates the species-specific binding to AMA1 Loop1E using genetically modified parasites and peptide inhibitors. We extended this knowledge by identifying specific amino acids that are critical for the species-specific divergence. Prior studies reported that a *Pv*RON2 peptide could bind recombinant *Pf*AMA1 protein with low affinity [22]. However, we found that *Pv*RON2 peptide could not inhibit the invasion of *P. falciparum* parasites expressing *Pf*AMA1. Structural analyses have found that RON2 binding causes conformational changes in AMA1. In its unbound state the hydrophobic cleft of AMA1 is partially covered by the disordered domain 2 loop. RON2 binding displaces the domain 2 loop [21, 54]. Some anti-*Pf*AMA1 mAbs, such as 1F9 and 4G2 [29, 55, 56], and those used in this study (4E11, 4E8, and 1B10), or anti-*Pk*AMA1 mAb R31C2 [21], are proposed to inhibit invasion by blocking the RON2 β-hairpin loop from binding the hydrophobic cleft of AMA1. In our study, we experimentally confirmed that three mAbs (4E11, 4E8 and 1B10) inhibit invasion by targeting Loop1E (Fig. 7C).

In conclusion, we used novel approaches to investigate AMA1–RON2 interactions in erythrocyte invasion in *P. falciparum* and *P. vivax*, the two major causes of malaria, with comparisons to *P. knowlesi*, a significant cause of disease in Southeast Asia. We defined species-specific AMA1–RON2 binding for invasive function in *P. vivax* versus *P. falciparum*, and cross-species conservation between *P. vivax* and *P. knowlesi*. Our data indicate substantial molecular flexibility in AMA1 interactions for invasion, which has interesting evolutionary implications, implying the adaptability of AMA1. High-affinity binding of the RON2 β-hairpin loop to AMA1 does not appear to be essential for invasion. Strategies to target this specific interaction have been a major focus of therapeutic and vaccine development, but strategies will need to be broader to include other AMA1 interactions to maximize efficacy. Our data also suggest that vaccine or therapeutic efficacy could be circumvented by mutations if targeting only the interaction between the RON2-loop and AMA1. The identification of specific residues that define invasion function and species-specific divergence or conservation will inform the design of novel vaccines and therapeutics against malaria.

## Supplementary Information

Below is the link to the electronic supplementary material.

Supplementary file1 (PDF 2180 KB)

## Data Availability

Data generated and analysed are available from the corresponding author on request. Unique reagents generated in this study are available from the corresponding author with a completed materials transfer agreement.

## Abbreviations

AMA1: Apical membrane protein 1
RON2: Rhoptry neck protein 2
RON2L: RON2-loop
*Pf*: *P. falciparum*
*Pv*: *P. vivax*
*Pk*: *P. knowlesi*
mAb: Monoclonal antibody

## References

[1] World Health Organization. World Malaria report 2021. Available from: https://www.who.int/publications/i/item/9789240040496. 2021.

[2] Mora C, McKenzie T, Gaw IM, Dean JM, von Hammerstein H, Knudson TA (2022). Over half of known human pathogenic diseases can be aggravated by climate change. Nat Clim Chang.

[3] Battle KE, Gething PW, Elyazar IR, Moyes CL, Sinka ME, Howes RE (2012). The global public health significance of *Plasmodium vivax*. Adv Parasitol.

[4] Beeson JG, Chu CS, Richards JS, Nosten F, Fowkes FJ (2015). *Plasmodium vivax* malaria: challenges in diagnosis, treatment and elimination. Pediatr Infect Dis J.

[5] Mueller I, Galinski MR, Baird JK, Carlton JM, Kochar DK, Alonso PL (2009). Key gaps in the knowledge of *Plasmodium vivax*, a neglected human malaria parasite. Lancet Infect Dis.

[6] Kho S, Qotrunnada L, Leonardo L, Andries B, Wardani PAI, Fricot A (2021). Hidden biomass of intact malaria parasites in the human spleen. N Engl J Med.

[7] Anstey NM, Grigg MJ, Rajahram GS, Cooper DJ, William T, Kho S (2021). Knowlesi malaria: human risk factors, clinical spectrum, and pathophysiology. Adv Parasitol.

[8] RTSS Clinical Trials Partnership (2015). Efficacy and safety of RTS, S/AS01 malaria vaccine with or without a booster dose in infants and children in Africa: final results of a phase 3, individually randomised, controlled trial. Lancet.

[9] Beeson JG, Kurtovic L, Dobano C, Opi DH, Chan JA, Feng G (2019). Challenges and strategies for developing efficacious and long-lasting malaria vaccines. Sci Transl Med..

[10] Tham WH, Beeson JG, Rayner JC (2017). *Plasmodium vivax* vaccine research—we've only just begun. Int J Parasitol.

[11] Beeson JG, Drew DR, Boyle MJ, Feng G, Fowkes FJ, Richards JS (2016). Merozoite surface proteins in red blood cell invasion, immunity and vaccines against malaria. FEMS Microbiol Rev.

[12] Silva JC, Egan A, Arze C, Spouge JL, Harris DG (2015). A new method for estimating species age supports the coexistence of malaria parasites and their Mammalian hosts. Mol Biol Evol.

[13] Taylor JE, Pacheco MA, Bacon DJ, Beg MA, Machado RL, Fairhurst RM (2013). The evolutionary history of *Plasmodium vivax* as inferred from mitochondrial genomes: parasite genetic diversity in the Americas. Mol Biol Evol.

[14] Lim C, Hansen E, DeSimone TM, Moreno Y, Junker K, Bei A (2013). Expansion of host cellular niche can drive adaptation of a zoonotic malaria parasite to humans. Nat Commun.

[15] Yap A, Azevedo MF, Gilson PR, Weiss GE, O'Neill MT, Wilson DW (2014). Conditional expression of apical membrane antigen 1 in *Plasmodium falciparum* shows it is required for erythrocyte invasion by merozoites. Cell Microbiol.

[16] Stanisic DI, Richards JS, McCallum FJ, Michon P, King CL, Schoepflin S (2009). Immunoglobulin G subclass-specific responses against *Plasmodium falciparum* merozoite antigens are associated with control of parasitemia and protection from symptomatic illness. Infect Immun.

[17] Polley SD, Mwangi T, Kocken CH, Thomas AW, Dutta S, Lanar DE (2004). Human antibodies to recombinant protein constructs of *Plasmodium falciparum* apical membrane antigen 1 (AMA1) and their associations with protection from malaria. Vaccine.

[18] Reiling L, Boyle MJ, White MT, Wilson DW, Feng G, Weaver R (2019). Targets of complement-fixing antibodies in protective immunity against malaria in children. Nat Commun.

[19] Thera MA, Doumbo OK, Coulibaly D, Laurens MB, Ouattara A, Kone AK (2011). A field trial to assess a blood-stage malaria vaccine. N Engl J Med.

[20] Parker ML, Boulanger MJ (2015). An extended surface loop on toxoplasma gondii apical membrane antigen 1 (AMA1) governs ligand binding selectivity. PLoS ONE.

[21] Vulliez-Le Normand B, Faber BW, Saul FA, van der Eijk M, Thomas AW, Singh B (2015). Crystal structure of *Plasmodium knowlesi* apical membrane antigen 1 and its complex with an invasion-inhibitory monoclonal antibody. PLoS ONE.

[22] Vulliez-Le Normand B, Saul FA, Hoos S, Faber BW, Bentley GA (2017). Cross-reactivity between apical membrane antgen 1 and rhoptry neck protein 2 in *P. vivax* and *P. falciparum*: a structural and binding study. PLoS ONE.

[23] Vulliez-Le Normand B, Tonkin ML, Lamarque MH, Langer S, Hoos S, Roques M (2012). Structural and functional insights into the malaria parasite moving junction complex. PLoS Pathog.

[24] Weiss GE, Gilson PR, Taechalertpaisarn T, Tham WH, de Jong NW, Harvey KL (2015). Revealing the sequence and resulting cellular morphology of receptor-ligand interactions during *Plasmodium falciparum* invasion of erythrocytes. PLoS Pathog.

[25] Besteiro S, Michelin A, Poncet J, Dubremetz JF, Lebrun M (2009). Export of a Toxoplasma gondii rhoptry neck protein complex at the host cell membrane to form the moving junction during invasion. PLoS Pathog.

[26] Lamarque M, Besteiro S, Papoin J, Roques M, Vulliez-Le Normand B, Morlon-Guyot J (2011). The RON2-AMA1 interaction is a critical step in moving junction-dependent invasion by apicomplexan parasites. PLoS Pathog.

[27] Tonkin ML, Roques M, Lamarque MH, Pugniere M, Douguet D, Crawford J (2011). Host cell invasion by apicomplexan parasites: insights from the co-structure of AMA1 with a RON2 peptide. Science.

[28] Lamarque MH, Roques M, Kong-Hap M, Tonkin ML, Rugarabamu G, Marq JB (2014). Plasticity and redundancy among AMA-RON pairs ensure host cell entry of Toxoplasma parasites. Nat Commun.

[29] Srinivasan P, Beatty WL, Diouf A, Herrera R, Ambroggio X, Moch JK (2011). Binding of *Plasmodium* merozoite proteins RON2 and AMA1 triggers commitment to invasion. Proc Natl Acad Sci U S A.

[30] Srinivasan P, Yasgar A, Luci DK, Beatty WL, Hu X, Andersen J (2013). Disrupting malaria parasite AMA1-RON2 interaction with a small molecule prevents erythrocyte invasion. Nat Commun.

[31] Drew DR, Sanders PR, Weiss G, Gilson PR, Crabb BS, Beeson JG (2018). Functional conservation of the AMA1 host-cell invasion ligand between *P. falciparum* and *P. vivax*: a novel platform to accelerate vaccine and drug development. J Infect Dis.

[32] Drew DR, Hodder AN, Wilson DW, Foley M, Mueller I, Siba PM (2012). Defining the antigenic diversity of *Plasmodium falciparum* apical membrane antigen 1 and the requirements for a multi-allele vaccine against malaria. PLoS ONE.

[33] Gruring C, Moon RW, Lim C, Holder AA, Blackman MJ, Duraisingh MT (2014). Human red blood cell-adapted *Plasmodium knowlesi* parasites: a new model system for malaria research. Cell Microbiol.

[34] Wright ES (2016). Using DECIPHER v2.0 to analyze big biological sequence data in R. R J.

[35] Brown SDJ, Collins RA, Boyer S, Lefort M-C, Malumbres-Olarte J, Vink CJ (2012). SPIDER: an R package for the analysis of species identity and evolution, with particular reference to DNA barcoding. Mol Ecol Resour.

[36] Boyle MJ, Reiling L, Feng G, Langer C, Osier FH, Aspeling-Jones H (2015). Human antibodies fix complement to inhibit *Plasmodium falciparum* invasion of erythrocytes and are associated with protection against malaria. Immunity.

[37] Boyle MJ, Wilson DW, Richards JS, Riglar DT, Tetteh KK, Conway DJ (2010). Isolation of viable *Plasmodium falciparum* merozoites to define erythrocyte invasion events and advance vaccine and drug development. Proc Natl Acad Sci U S A.

[38] Drew DR, Wilson DW, Elliott SR, Cross N, Terheggen U, Hodder AN (2016). A novel approach to identifying patterns of human invasion-inhibitory antibodies guides the design of malaria vaccines incorporating polymorphic antigens. BMC Med.

[39] Kurtovic L, Agius PA, Feng G, Drew DR, Ubillos I, Sacarlal J (2019). Induction and decay of functional complement-fixing antibodies by the RTS, S malaria vaccine in children, and a negative impact of malaria exposure. BMC Med.

[40] Kurtovic L, Drew DR, Dent AE, Kazura JW, Beeson JG (2021). Antibody targets and properties for complement-fixation against the circumsporozoite protein in malaria immunity. Front Immunol.

[41] Dutta S, Dlugosz LS, Drew DR, Ge X, Ababacar D, Rovira YI (2013). Overcoming antigenic diversity by enhancing the immunogenicity of conserved epitopes on the malaria vaccine candidate apical membrane antigen-1. PLoS Pathog.

[42] Terheggen U, Drew DR, Hodder AN, Cross NJ, Mugyenyi CK, Barry AE (2014). Limited antigenic diversity of *Plasmodium falciparum* apical membrane antigen 1 supports the development of effective multi-allele vaccines. BMC Med.

[43] Persson KE, McCallum FJ, Reiling L, Lister NA, Stubbs J, Cowman AF (2008). Variation in use of erythrocyte invasion pathways by *Plasmodium falciparum* mediates evasion of human inhibitory antibodies. J Clin Invest.

[44] Wilson DW, Crabb BS, Beeson JG (2010). Development of fluorescent *Plasmodium falciparum* for in vitro growth inhibition assays. Malar J.

[45] Persson KE, Lee CT, Marsh K, Beeson JG (2006). Development and optimization of high-throughput methods to measure *Plasmodium falciparum*-specific growth inhibitory antibodies. J Clin Microbiol.

[46] Narum DL, Thomas AW (1994). Differential localization of full-length and processed forms of PF83/AMA-1 an apical membrane antigen of *Plasmodium falciparum* merozoites. Mol Biochem Parasitol.

[47] Arevalo-Pinzon G, Bermudez M, Hernandez D, Curtidor H, Patarroyo MA (2017). *Plasmodium vivax* ligand-receptor interaction: PvAMA-1 domain I contains the minimal regions for specific interaction with CD71+ reticulocytes. Sci Rep.

[48] Kato K, Mayer DC, Singh S, Reid M, Miller LH (2005). Domain III of *Plasmodium falciparum* apical membrane antigen 1 binds to the erythrocyte membrane protein Kx. Proc Natl Acad Sci U S A.

[49] Treeck M, Zacherl S, Herrmann S, Cabrera A, Kono M, Struck NS (2009). Functional analysis of the leading malaria vaccine candidate AMA-1 reveals an essential role for the cytoplasmic domain in the invasion process. PLoS Pathog.

[50] Leykauf K, Treeck M, Gilson PR, Nebl T, Braulke T, Cowman AF (2010). Protein kinase a dependent phosphorylation of apical membrane antigen 1 plays an important role in erythrocyte invasion by the malaria parasite. PLoS Pathog.

[51] Singh S, Alam MM, Pal-Bhowmick I, Brzostowski JA, Chitnis CE (2010). Distinct external signals trigger sequential release of apical organelles during erythrocyte invasion by malaria parasites. PLoS Pathog.

[52] Dicko A, Diemert DJ, Sagara I, Sogoba M, Niambele MB, Assadou MH (2007). Impact of a *Plasmodium falciparum* AMA1 vaccine on antibody responses in adult Malians. PLoS ONE.

[53] Sagara I, Ellis RD, Dicko A, Niambele MB, Kamate B, Guindo O (2009). A randomized and controlled Phase 1 study of the safety and immunogenicity of the AMA1-C1/Alhydrogel + CPG 7909 vaccine for *Plasmodium falciparum* malaria in semi-immune Malian adults. Vaccine.

[54] Delgadillo RF, Parker ML, Lebrun M, Boulanger MJ, Douguet D (2016). Stability of the *Plasmodium falciparum* AMA1-RON2 Complex Is Governed by the Domain II (DII) Loop. PLoS ONE.

[55] Coley AM, Parisi K, Masciantonio R, Hoeck J, Casey JL, Murphy VJ (2006). The most polymorphic residue on *Plasmodium falciparum* apical membrane antigen 1 determines binding of an invasion-inhibitory antibody. Infect Immun.

[56] Collins CR, Withers-Martinez C, Hackett F, Blackman MJ (2009). An inhibitory antibody blocks interactions between components of the malarial invasion machinery. PLoS Pathog.

