# Supplementary material for: Defining species-specific and conserved interactions of apical membrane protein 1 during erythrocyte invasion in malaria to inform multi-species vaccines
Source: Cell Mol Life Sci. 2023 Feb 27;80(3):74. doi: 10.1007/s00018-023-04712-z (PMC9969379; doi:10.1007/s00018-023-04712-z)
Supplement: Supplementary file 1 — Supplementary file1 (PDF 2180 KB) [file 18_2023_4712_MOESM1_ESM.pdf]

SUPPLEMENTARY MATERIAL

Drew DR, et al

Defining species-specific and conserved interactions of apical membrane protein 1 during erythrocyte invasion in malaria to inform multi-species vaccines

Full length Ron2 amino acid sequence identity matrix

	P.gallinace...	P.relictum	P.facliparum	P.reichenowi	P.coatneyi	P.knowlesi	P.vivax	P.cynomogi	P.ovale	P.malariae	P.berghei	P.yoelii	P.chabaudi	B.bovis	T.gondii
P.gallinaceum		63.499%	42.416%	43.011%	42.380%	42.373%	41.190%	51.961%	44.482%	37.614%	36.460%	35.600%	36.929%	20.355%	14.022%
P.relictum	63.499%		43.878%	44.321%	43.036%	42.499%	41.625%	46.987%	43.420%	37.904%	35.721%	35.505%	36.220%	20.708%	14.642%
P.facliparum	42.416%	43.878%		89.910%	43.651%	43.896%	43.356%	41.085%	45.269%	40.112%	36.708%	36.583%	36.168%	19.166%	14.796%
P.reichenowi	43.011%	44.321%	89.910%		44.220%	44.335%	43.548%	41.632%	45.605%	40.337%	37.354%	37.226%	37.037%	19.083%	14.899%
P.coatneyi	42.380%	43.036%	43.651%	44.220%		85.390%	81.946%	64.228%	59.026%	49.550%	39.140%	39.279%	37.977%	20.621%	14.496%
P.knowlesi	42.373%	42.499%	43.896%	44.335%	85.390%		80.616%	63.989%	59.972%	50.205%	39.178%	39.471%	38.069%	20.555%	14.687%
P.vivax	41.190%	41.625%	43.356%	43.548%	81.946%	80.616%		63.603%	58.669%	50.123%	38.667%	38.530%	37.159%	20.979%	14.450%
P.cynomogi	51.961%	46.987%	41.085%	41.632%	64.228%	63.989%	63.603%		53.285%	43.837%	38.144%	37.715%	37.339%	20.423%	14.815%
P.ovale	44.482%	43.420%	45.269%	45.605%	59.026%	59.972%	58.669%	53.285%		52.037%	40.075%	40.103%	38.892%	20.662%	14.139%
P.malariae	37.614%	37.904%	40.112%	40.337%	49.550%	50.205%	50.123%	43.837%	52.037%		34.315%	34.545%	34.008%	19.562%	13.892%
P.berghei	36.460%	35.721%	36.708%	37.354%	39.140%	39.178%	38.667%	38.144%	40.075%	34.315%		80.753%	69.407%	19.264%	13.118%
P.yoelii	35.600%	35.505%	36.583%	37.226%	39.279%	39.471%	38.530%	37.715%	40.103%	34.545%	80.753%		66.930%	19.321%	13.359%
P.chabaudi	36.929%	36.220%	36.168%	37.037%	37.977%	38.069%	37.159%	37.339%	38.892%	34.008%	69.407%	66.930%		20.340%	13.911%
B.bovis	20.355%	20.708%	19.166%	19.083%	20.621%	20.555%	20.979%	20.423%	20.662%	19.562%	19.264%	19.321%	20.340%		13.002%
T.gondii	14.022%	14.642%	14.796%	14.899%	14.496%	14.687%	14.450%	14.815%	14.139%	13.892%	13.118%	13.359%	13.911%	13.002%	

Full length Ron2 amino acid sequence similarity matrix

	P.gallinace...	P.relictum	P.facliparum	P.reichenowi	P.coatneyi	P.knowlesi	P.vivax	P.cynomogi	P.ovale	P.malariae	P.berghei	P.yoelii	P.chabaudi	B.bovis	T.gondii
P.gallinaceum		79.542%	61.918%	63.227%	61.665%	61.864%	59.863%	72.030%	63.795%	54.267%	59.307%	58.357%	60.512%	58.265%	45.095%
P.relictum	79.542%		69.049%	70.468%	67.734%	67.627%	65.553%	67.413%	67.483%	58.043%	64.231%	63.751%	66.093%	57.698%	45.634%
P.facliparum	61.918%	69.049%		93.094%	71.914%	71.617%	70.492%	61.397%	70.484%	63.477%	65.053%	65.130%	64.145%	58.574%	47.047%
P.reichenowi	63.227%	70.468%	93.094%		72.849%	71.989%	70.619%	62.588%	71.284%	63.232%	66.607%	66.040%	65.661%	58.328%	46.975%
P.coatneyi	61.665%	67.734%	71.914%	72.849%		94.991%	91.765%	72.597%	81.002%	69.951%	66.712%	67.108%	65.379%	56.709%	46.169%
P.knowlesi	61.864%	67.627%	71.617%	71.989%	94.991%		91.712%	72.501%	82.004%	70.131%	67.489%	67.685%	65.839%	57.105%	46.360%
P.vivax	59.863%	65.553%	70.492%	70.619%	91.765%	91.712%		70.984%	80.074%	70.867%	65.733%	66.013%	63.421%	56.122%	45.972%
P.cynomogi	72.030%	67.413%	61.397%	62.588%	72.597%	72.501%	70.984%		69.586%	58.982%	60.933%	60.325%	60.386%	56.775%	46.105%
P.ovale	63.795%	67.483%	70.484%	71.284%	81.002%	82.004%	80.074%	69.586%		70.685%	67.121%	67.936%	65.372%	56.887%	46.385%
P.malariae	54.267%	58.043%	63.477%	63.232%	69.951%	70.131%	70.867%	58.982%	70.685%		59.435%	60.323%	57.409%	57.361%	46.159%
P.berghei	59.307%	64.231%	65.053%	66.607%	66.712%	67.489%	65.733%	60.933%	67.121%	59.435%		90.051%	85.707%	54.832%	44.233%
P.yoelii	58.357%	63.751%	65.130%	66.040%	67.108%	67.685%	66.013%	60.325%	67.936%	60.323%	90.051%		82.605%	54.961%	44.802%
P.chabaudi	60.512%	66.093%	64.145%	65.661%	65.379%	65.839%	63.421%	60.386%	65.372%	57.409%	85.707%	82.605%		56.667%	45.538%
B.bovis	58.265%	57.698%	58.574%	58.328%	56.709%	57.105%	56.122%	56.775%	56.887%	57.361%	54.832%	54.961%	56.667%		43.363%
T.gondii	45.095%	45.634%	47.047%	46.975%	46.169%	46.360%	45.972%	46.105%	46.385%	46.159%	44.233%	44.802%	45.538%	43.363%	

Figure S1: Ron2 protein amino acid sequence identity matrix and similarity matrix

Ron2 peptide amino acid sequence alignment

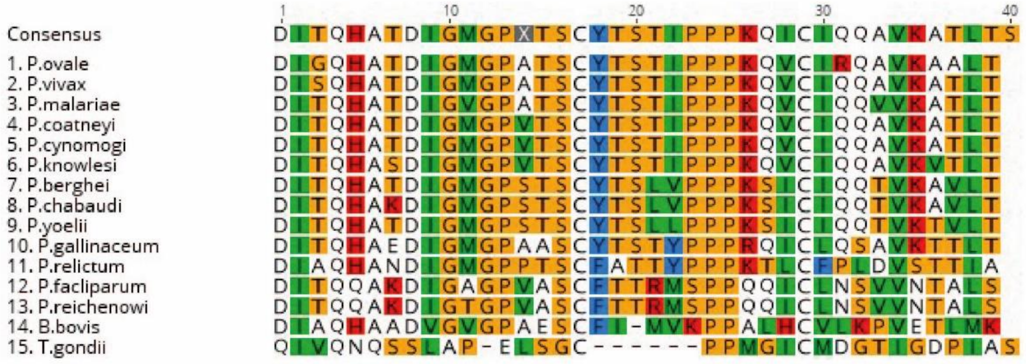

Ron2 peptide amino acid sequence identity matrix

	P.ovale	P.vivax	P.malariae	P.coatneyi	P.cynomogi	P.knowlesi	P.berghei	P.chabaudi	P.yoelii	P.gallinace...	P.relictum	P.facliparum	P.reichenowi	B.bovis	T.gondii
P.ovale		92.308%	87.179%	89.744%	89.744%	84.615%	76.923%	74.359%	74.359%	71.795%	53.846%	48.718%	48.718%	41.026%	15.385%
P.vivax	92.308%		92.308%	94.872%	94.872%	89.744%	79.487%	76.923%	76.923%	76.923%	56.410%	46.154%	46.154%	41.026%	15.385%
P.malariae	87.179%	92.308%		92.308%	92.308%	87.179%	79.487%	76.923%	76.923%	74.359%	53.846%	51.282%	51.282%	43.590%	15.385%
P.coatneyi	89.744%	94.872%	92.308%		100%	94.872%	82.051%	79.487%	79.487%	76.923%	56.410%	51.282%	51.282%	38.462%	15.385%
P.cynomogi	89.744%	94.872%	92.308%	100%		94.872%	82.051%	79.487%	79.487%	76.923%	56.410%	51.282%	51.282%	38.462%	15.385%
P.knowlesi	84.615%	89.744%	87.179%	94.872%	94.872%		76.923%	76.923%	76.923%	76.923%	56.410%	51.282%	51.282%	38.462%	17.949%
P.berghei	76.923%	79.487%	79.487%	82.051%	82.051%	76.923%		97.436%	94.872%	69.231%	51.282%	48.718%	48.718%	41.026%	20.513%
P.chabaudi	74.359%	76.923%	76.923%	79.487%	79.487%	76.923%	97.436%		92.308%	69.231%	51.282%	51.282%	51.282%	41.026%	20.513%
P.yoelii	74.359%	76.923%	76.923%	79.487%	79.487%	76.923%	94.872%	92.308%		71.795%	53.846%	51.282%	51.282%	41.026%	20.513%
P.gallinaceum	71.795%	76.923%	74.359%	76.923%	76.923%	76.923%	69.231%	69.231%	71.795%		56.410%	61.538%	61.538%	43.590%	17.949%
P.relictum	53.846%	56.410%	53.846%	56.410%	56.410%	56.410%	51.282%	51.282%	53.846%	56.410%		46.154%	46.154%	46.154%	20.513%
P.facliparum	48.718%	46.154%	51.282%	51.282%	51.282%	51.282%	48.718%	51.282%	51.282%	61.538%	46.154%		97.436%	41.026%	17.949%
P.reichenowi	48.718%	46.154%	51.282%	51.282%	51.282%	51.282%	48.718%	51.282%	51.282%	61.538%	46.154%	97.436%		41.026%	17.949%
B.bovis	41.026%	41.026%	43.590%	38.462%	38.462%	38.462%	41.026%	41.026%	41.026%	43.590%	46.154%	41.026%	41.026%		15.789%
T.gondii	15.385%	15.385%	15.385%	15.385%	15.385%	17.949%	20.513%	20.513%	20.513%	17.949%	20.513%	17.949%	17.949%	15.789%	

Ron2 peptide amino acid sequence difference matrix

	P.ovale	P.vivax	P.malariae	P.coatneyi	P.cynomogi	P.knowlesi	P.berghei	P.chabaudi	P.yoelii	P.gallinace...	P.relictum	P.facliparum	P.reichenowi	B.bovis	T.gondii
P.ovale		3	5	4	4	6	9	10	10	11	18	20	20	23	33
P.vivax	3		3	2	2	4	8	9	9	9	17	21	21	23	33
P.malariae	5	3		3	3	5	8	9	9	10	18	19	19	22	33
P.coatneyi	4	2	3		0	2	7	8	8	9	17	19	19	24	33
P.cynomogi	4	2	3	0		2	7	8	8	9	17	19	19	24	33
P.knowlesi	6	4	5	2	2		9	9	9	9	17	19	19	24	32
P.berghei	9	8	8	7	7	9		1	2	12	19	20	20	23	31
P.chabaudi	10	9	9	8	8	9	1		3	12	19	19	19	23	31
P.yoelii	10	9	9	8	8	9	2	3		11	18	19	19	23	31
P.gallinaceum	11	9	10	9	9	9	12	12	11		17	15	15	22	32
P.relictum	18	17	18	17	17	17	19	19	18	17		21	21	21	31
P.facliparum	20	21	19	19	19	19	20	19	19	15	21		1	23	32
P.reichenowi	20	21	19	19	19	19	20	19	19	15	21	1		23	32
B.bovis	23	23	22	24	24	24	23	23	23	22	21	23	23		32
T.gondii	33	33	33	33	33	32	31	31	31	32	31	32	32	32	

Figure S2: Ron2 peptide amino acid sequence alignments, identity matrix and amino acid difference matrix.

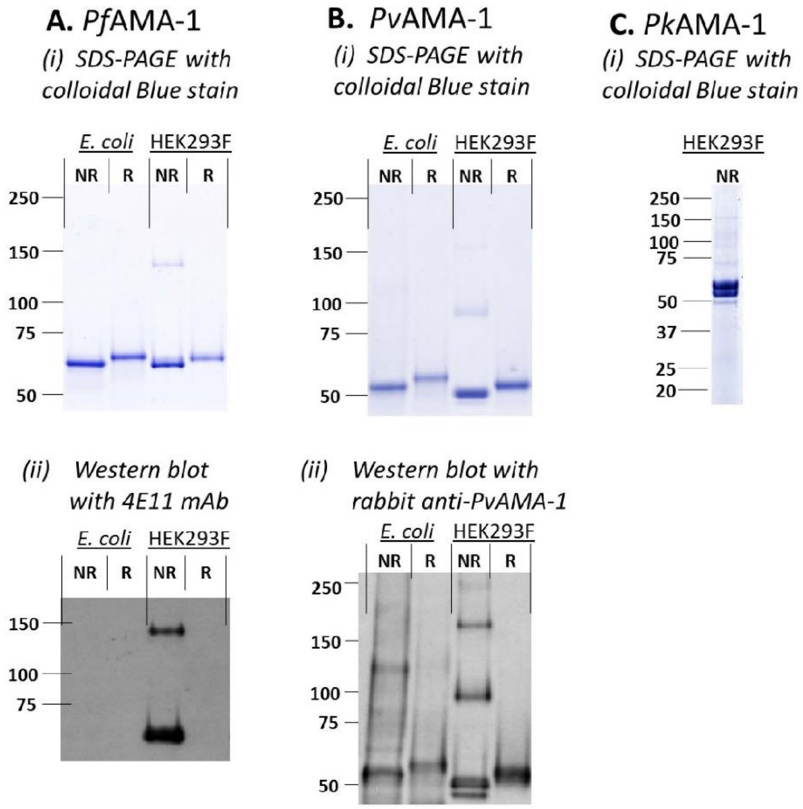

Figure S3. (A) *Pf*AMA-1, (B) *Pv*AMA-1 and (C) *Pk*AMA-1 protein. The purified recombinant proteins (1 µg) were run on an SDS-PAGE gel and then tested for protein presence using a (i) protein gel (stained with colloidal blue) or antibody recognition by (ii) western blot. (A-i) *Pf*AMA-1, and (B-i) *Pv*AMA-1 were tested as reduced (TCEP) and non-reduced and compared to *E. coli* expressed protein. (A-ii) *Pf*AMA1 was also tested for binding of the 4E11 mAb and (B-ii) polyclonal rabbit antibodies. (C) *Pk*AMA-1 was tested as a non-reduced protein.

AMA1 Loop 1E amino acid sequence alignment: extracted from *P.falciparum* AMA1 homologues

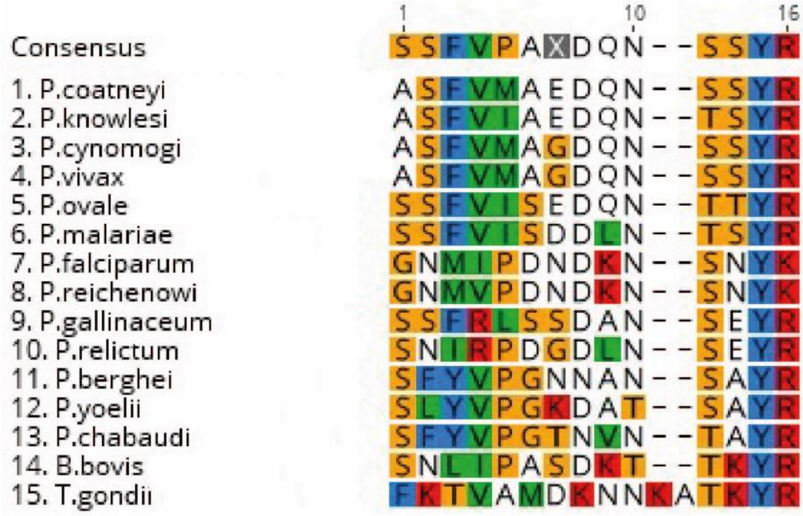

AMA1 Loop 1E amino acid sequence identity matrix

	P.coatneyi ...	P.knowlesi...	P.cynomog...	P.vivax ext...	P.ovale e...	P.malariae...	P.falciparu...	P.reicheno...	P.gallinace...	P.relictum ...	P.berghei ...	P.yoelii ex...	P.chabaud...	B.bovis ex...	T.gondii e...
P.coatneyi		85.714%	92.857%	92.857%	64.286%	57.143%	28.571%	35.714%	50.000%	35.714%	35.714%	35.714%	28.571%	28.571%	25.000%
P.knowlesi	85.714%		78.571%	78.571%	78.571%	71.429%	21.429%	28.571%	42.857%	28.571%	28.571%	28.571%	35.714%	35.714%	31.250%
P.cynomogi	92.857%	78.571%		100%	57.143%	57.143%	28.571%	35.714%	50.000%	42.857%	35.714%	35.714%	28.571%	28.571%	25.000%
P.vivax	92.857%	78.571%	100%		57.143%	57.143%	28.571%	35.714%	50.000%	42.857%	35.714%	35.714%	28.571%	28.571%	25.000%
P.ovale	64.286%	78.571%	57.143%	57.143%		78.571%	21.429%	28.571%	57.143%	35.714%	35.714%	35.714%	42.857%	35.714%	31.250%
P.malariae	57.143%	71.429%	57.143%	57.143%	78.571%		21.429%	28.571%	57.143%	42.857%	35.714%	35.714%	42.857%	35.714%	37.500%
P.falciparum	28.571%	21.429%	28.571%	28.571%	21.429%	21.429%		92.857%	28.571%	50.000%	35.714%	28.571%	21.429%	42.857%	12.500%
P.reichenowi	35.714%	28.571%	35.714%	35.714%	28.571%	28.571%	92.857%		28.571%	50.000%	42.857%	35.714%	28.571%	35.714%	18.750%
P.gallinaceum	50.000%	42.857%	50.000%	50.000%	57.143%	57.143%	28.571%	28.571%		57.143%	42.857%	42.857%	28.571%	35.714%	18.750%
P.relictum	35.714%	28.571%	42.857%	42.857%	35.714%	42.857%	50.000%	50.000%	57.143%		42.857%	42.857%	35.714%	42.857%	18.750%
P.berghei	35.714%	28.571%	35.714%	35.714%	35.714%	35.714%	35.714%	42.857%	42.857%	42.857%		71.429%	78.571%	28.571%	25.000%
P.yoelii	35.714%	28.571%	35.714%	35.714%	35.714%	35.714%	28.571%	35.714%	42.857%	42.857%	71.429%		57.143%	42.857%	18.750%
P.chabaudi	28.571%	35.714%	28.571%	28.571%	42.857%	42.857%	21.429%	28.571%	28.571%	35.714%	78.571%	57.143%		35.714%	31.250%
B.bovis	28.571%	35.714%	28.571%	28.571%	35.714%	35.714%	42.857%	35.714%	35.714%	42.857%	28.571%	42.857%	35.714%		25.000%
T.gondii	25.000%	31.250%	25.000%	25.000%	31.250%	37.500%	12.500%	18.750%	18.750%	18.750%	25.000%	18.750%	31.250%	25.000%	

AMA1 Loop 1E amino acid sequence difference matrix

	P.coatneyi ...	P.knowlesi...	P.cynomog...	P.vivax ext...	P.ovale e...	P.malariae...	P.falciparu...	P.reicheno...	P.gallinace...	P.relictum ...	P.berghei ...	P.yoelii ex...	P.chabaud...	B.bovis ex...	T.gondii e...
P.coatneyi		2	1	1	5	6	10	9	7	9	9	9	10	10	12
P.knowlesi	2		3	3	3	4	11	10	8	10	10	10	9	9	11
P.cynomogi	1	3		0	6	6	10	9	7	8	9	9	10	10	12
P.vivax	1	3	0		6	6	10	9	7	8	9	9	10	10	12
P.ovale	5	3	6	6		3	11	10	6	9	9	9	8	9	11
P.malariae	6	4	6	6	3		11	10	6	8	9	9	8	9	10
P.falciparum	10	11	10	10	11	11		1	10	7	9	10	11	8	14
P.reichenowi	9	10	9	9	10	10	1		10	7	8	9	10	9	13
P.gallinaceum	7	8	7	7	6	6	10	10		6	8	8	10	9	13
P.relictum	9	10	8	8	9	8	7	7	6		8	8	9	8	13
P.berghei	9	10	9	9	9	9	9	8	8	8		4	3	10	12
P.yoelii	9	10	9	9	9	9	10	9	8	8	4		6	8	13
P.chabaudi	10	9	10	10	8	8	11	10	10	9	3	6		9	11
B.bovis	10	9	10	10	9	9	8	9	9	8	10	8	9		12
T.gondii	12	11	12	12	11	10	14	13	13	13	12	13	11	12	

Figure S4: AMA1 Loop1E amino acid sequence alignments, identity matrix and amino acid difference matrix.

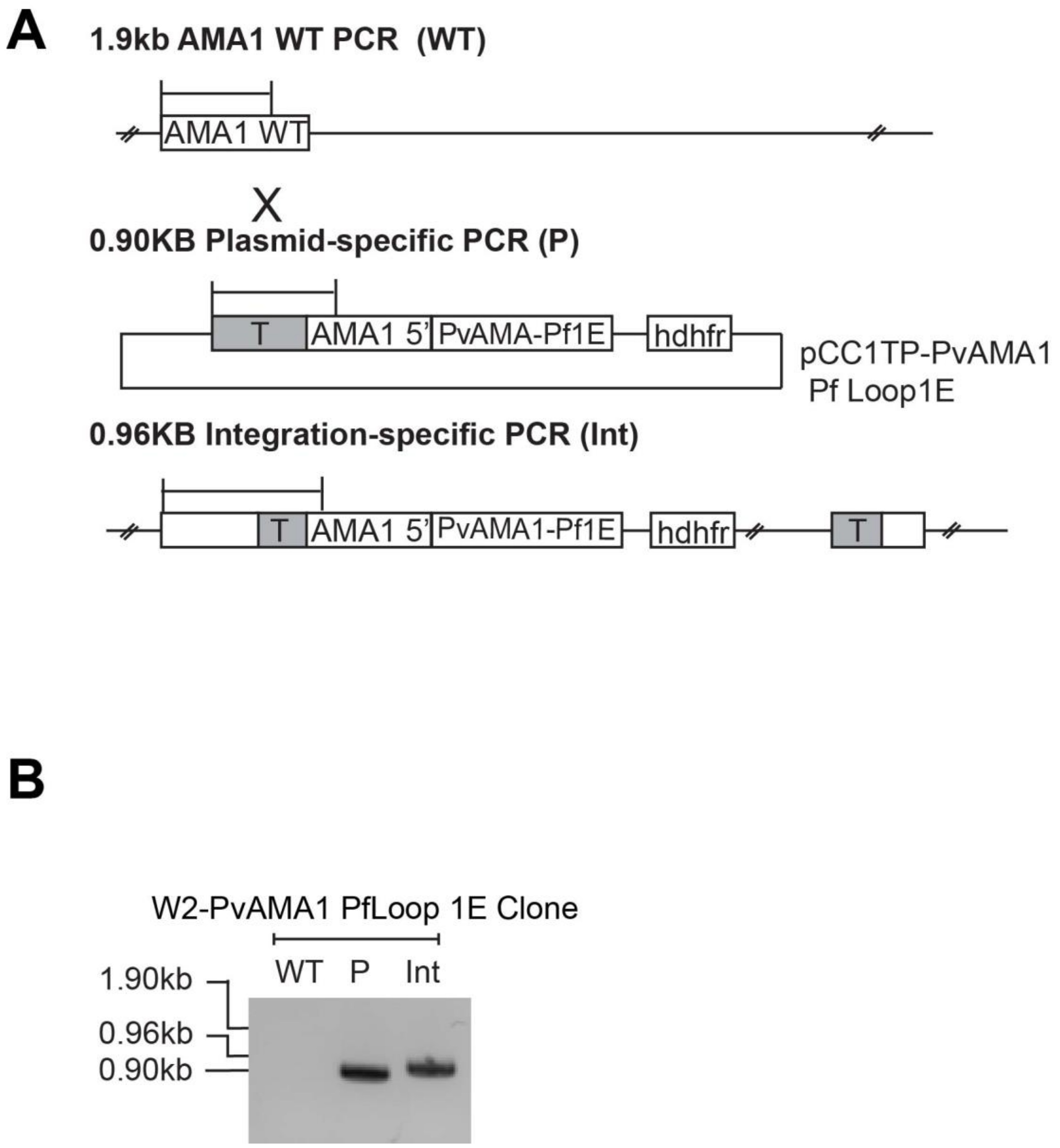

Figure S5: Generation of transgenic *P. falciparum* that express chimeric PvAMA1 incorporating *P. falciparum* Loop1E amino acids. **A.** Plasmid design and integration. A codon optimised PvAMA1 gene incorporating *P .falciparum* 3D7 PfAMA1 Loop 1E amino acid substitutions at positions 222-228 was transfected into W2Mef parental parasites. The single-crossover event for allelic replacement of the wild type (WT) AMA1 is illustrated. **B.** PCR confirmation of allelic exchange of the PvAMA1 PfLoop1E gene into the WT AMA1 locus. The regions amplified by PCR are indicated by horizontal lines above the DNA schematics in (A). The expected sizes for wild type (WT), non-integrated plasmid (P) and integrated 3D7 AMA1 (Int) are shown in kilobases (kb). Transgenic parasites were screened for the presence of the wild-type locus, transfection plasmid, and integrated plasmid. The first lane shows an absence of the wild-type locus (WT, expected size 1.90 kb). The second lane shows the presence of the transfection plasmid (P, expected size 0.90 kb), which has integrated into the parasites' genome (third lane, Int, expected size 0.96 kb).

A

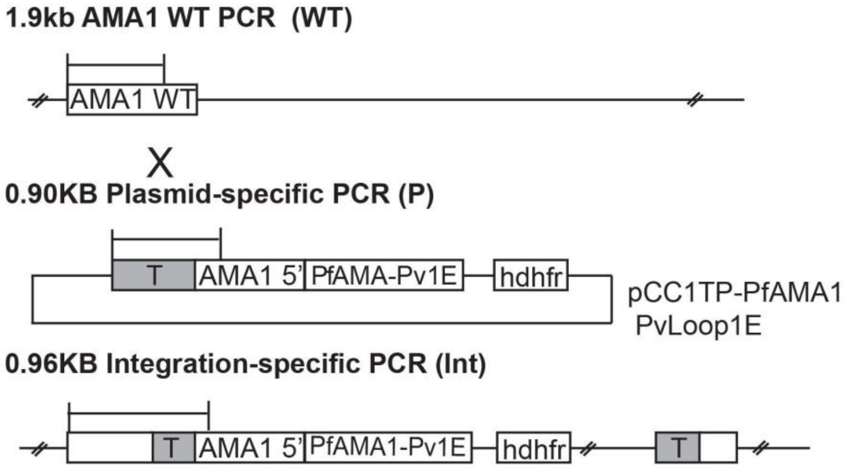

B

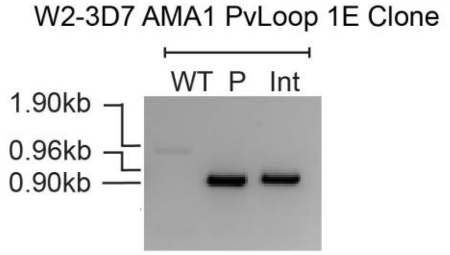

Figure S6: Generation of transgenic *P .falciparum* that expresses chimeric 3D7 AMA1 incorporating *P .vivax* Loop1E amino acids**. A.** Plasmid design and integration. A codon optimised 3D7 PfAMA1 gene incorporating *P.vivax* AMA1 Loop 1E amino acid substitutions at positions 222-228 was transfected into W2Mef parental parasites. The single-crossover event for allelic replacement of the wild type (WT) AMA1 is illustrated. **B.** PCR confirmation of allelic exchange of the PfAMA1 PvLoop1E gene into the WT AMA1 locus (W2-3D7 AMA1 PvLoop1E Clone). The regions amplified by PCR are indicated by horizontal lines above the DNA schematics in (A). The expected sizes for wild type (WT), non-integrated plasmid (P) and integrated 3D7 PfAMA1 (Int) are shown in kilobases (kb). Transgenic parasites were screened for the presence of the wild-type locus, transfection plasmid, and integrated plasmid. The first lane shows lack of detection of the wild-type locus (WT, expected size 1.90 kb). The second lane shows the presence of the transfection plasmid (P, expected size 0.90 kb), which has integrated into the parasite genome (third lane, Int, expected size 0.96 kb).

Alignment of AMA-1 sequences from *P. falciparum*, *P. vivax* and *P. knowlesi*

```
PfAMA-1 MDAMKRGLCCVLLLCGAVFVSASHHHHHHQNYWEHPYQNSDVYRPINEHREHPKEYEYPL
PvAMA-1 MDAMKRGLCCVLLLCGAVFVSASHHHHHH-------------------------------
PkAMA-1 MDAMKRGLCCVLLLCGAVFVSASHHHHHH-------------------------------
        *****************************###############################

PfAMA-1 HQEHTYQQEDSGEDENTLQHAYPIDHEGAEPAPQEQNLFSSIEIVERSNYMGNPWTEYMA
PvAMA-1 -----------GRNQKPSRLTRSANNVLLEKGP----------TVERSTRMSNPWKAFME
PkAMA-1 -----------ERNQKTTRLTRSANNALLEKGP----------IIERSIRMSNPWKAFME
        #############:::.#.#:#.#::###*#.*###########:***##*.***.#:*#

                                                    !
PfAMA-1 KYDIEEVHGSGIRVDLGEDAEVAGTQYRLPSGKCPVFGKGIIIENSKTTFLTPVATGNQY
PvAMA-1 KYDIERTHSSGVRVDLGEDAEVENAKYRIPAGRCPVFGKGIVIENSAVSFLKPVATGDQR
PkAMA-1 KYDLERAHNSGIRIDLGEDAEVGNSKYRIPAGKCPVFGKGIVIENSAVSFLTPVATGAQR
        ***:*#.*.**:*:********#.::**:*:*.********:****#.:**.*****#*#

PfAMA-1 LKDGGFAFPPTEPLMSPMTLDEMRHFYKDNKYVKNLDELTLCSRHAGNMIPDNDKNSNYK
PvAMA-1 LKDGGFAFPNANDHISPMTLENLKARYKDNVEMMKLNDIALCRTHAASFVMAGDQNSNYR
PkAMA-1 LKEGGFAFPNADDHISPITIANLKERYKENADLMKLNDIALCKTHAASFVIAEDQNSSYR
        **:******#::##:**:*:#::.##**:*##:#:*::::**##**..::###*:**.*.

PfAMA-1 YPAVYDDKDKKCHILYIAAQENNGPRYCNKDESKRNSMFCFRPAKDISFQNYVYLSKNVV
PvAMA-1 HPAVYDEKEKTCHMLYLSAQENMGPRYCSPDAQNRDAVFCFKPDKDESFENLVYLSKNVR
PkAMA-1 HPAVYDEKEKTCYMLYLSAQENMGPRYCSPDSQNKDAMFCFKPDKNEKFDNLVYLSKNSR
        :*****:*:*.*::**::****#******.#*#.:.:::***.*#*:#.*:*#******##

PfAMA-1 DNWEKVCPRKNLQNAKFGLWVDGNCEDIPHVNEFPAIDLFECNKLVFELSASDQPKQYEQ
PvAMA-1 NDWDKKCPRKNLGNAKFGLWVDGNCEEIPYVKEVEAKDLRECNRIVFEASASDQPTQYEE
PkAMA-1 NDWENKCPRKNLGNAKFGLWVDGNCEEIPYVNEVEARSLRECNRIVFEASASDQPRQYEE
        ::*::#******#*************:**:*:*.#*#.*#***.:***#******#***:

PfAMA-1 HLTDYEKIKEGFKNKNADMIKSAFLPTGAFKADRYKSHGKGYNWGNYNTETQKCEIFNVK
PvAMA-1 EMTDYQKIQQGFRQNNREMIKSAFLPVGAFNSDNFKSKGRGFNWANFDSVKKKCYIFNTK
PkAMA-1 ELTDYEKIQEGFRQNNRDMIKSAFLPVGAFNSDNFKSKGRGYNWANFDSVNNKCYIFNTK
        #:***:**::**.::*#:*********.***::*.:**:*.*:**.*:::#.:**#***.*

PfAMA-1 PTCLINSKSYIATTALSHPIEVENNFPCSLYKDEIMKEIERESKRIKLNDNDDEGNKKII
PvAMA-1 PTCLINDKNFIATTALSHPREVDLEFPCSIYKDEIEREIKKQSRNMNLYSVDGE---RIV
PkAMA-1 PTCLINDKNFFATTALSHPQEVDNEFPCSIYKDEIEREIKKQSRNMNLYSVDKE---RIV
        ******.*.::********* **:#:****:*****#.**:.:*..::*#.#*#*###.*:

PfAMA-1 APRIFISDDKDSLKCPCDPEMVSESTCRFFVCKCVERRAEVTSNNEVVVKEEYKDEYADI
PvAMA-1 LPRIFISNDKESIKCPCEPEHISQSTCNFYVCNCVEKRAEIKENNQVVIKEEFRDYYEN-
PkAMA-1 LPRIFISTDKESIKCPCEPEHISQSTCNFYVCNCVEKRAEIKENNEVIIKEEFKEDYEN-
        #******#**:*:****:**#:*:***.*:**:***.***:..**:*::***:.:#*#:#

PfAMA-1 PEHKPTYDKMK
PvAMA-1 GEEK---SNKQ
PkAMA-1 PDGK---HKKK
        #:#*####:#:
```

Figure S7: Alignment of the PfAMA1, PvAMA1 and PkAMA1 amino acid sequences expressed as recombinant proteins in HEK293F cells

Table 1. Details of the sequences used to make the recombinant AMA-1						
Antigen	Species	Strain	Accession no.	Expressed aa	Length (aa)	Modified to remove glycosylation motif?
AMA-1	*P. falciparum*	3D7	XP_001348015.1<br>PF11_0344	Q25-K546	522	Yes, Table 2
	P. vivax	Palo Alto	ACB42433.1<br>EU395595.1	G22-Q484	463	Yes, Table 2
	P. knowlesi	H	XP_002259339.1<br>PKH_093110	E22-K484	463	Yes, Table 2

Table 2. Modifications to the AMA-1 protein sequence to remove potential glycosylation sites.

	Potential N-glycosylation		Modifications to remove potential glycosylation sites	
	Amino acid no.	Amino acids	Modified amino acids	Modified seq.
Pf AMA-1	162	NTT	162 N-K	KTT
	286	NYT	288 T-V	NYV
	371	NAS	373 S-D	NAD
	421<br>422	NNSS	422 N-S<br>423 S-K	NSKS
	499	NST	499 N-E	EST
Pv AMA-1	176	NSS	178 S-N	NSN
	226	NES	226 N-D	DES
	441	NST	441 N-Q	QST
Pk AMA-1	36	NAS	38 S-L	NAL
	107	NVS	107 N-A	AVS
	176	NTS	177 T-S	NSS
	189	NKT	189 N-E	EKT
	238	NVS	239 V-S<br>240 S-R	NSR
	441	NST	441 N-Q	QST
